# CD73 promotes hepatocellular carcinoma progression and metastasis via activating PI3K/AKT signaling by inducing Rap1-mediated membrane localization of P110β and predicts poor prognosis

**DOI:** 10.1186/s13045-019-0724-7

**Published:** 2019-04-11

**Authors:** Xiao-Lu Ma, Min-Na Shen, Bo Hu, Bei-Li Wang, Wen-Jing Yang, Li-Hua Lv, Hao Wang, Yan Zhou, An-Li Jin, Yun-Fan Sun, Chuan-Yan Zhang, Shuang-Jian Qiu, Bai-Shen Pan, Jian Zhou, Jia Fan, Xin-Rong Yang, Wei Guo

**Affiliations:** 1 Department of Laboratory Medicine, Zhongshan Hospital, Fudan University, 136 Yi Xue Yuan Road, Shanghai, 200032 People’s Republic of China; 2 Department of Liver Surgery, Liver Cancer Institute, Zhongshan Hospital, Fudan University, Key Laboratory of Carcinogenesis and Cancer Invasion, Ministry of Education, Shanghai, 200032 People’s Republic of China; 3 Liver Cancer Institute, Fudan University, 136 Yi Xue Yuan Road, Shanghai, 200032 People’s Republic of China

**Keywords:** Hepatocellular carcinoma, CD73, Epithelial-mesenchymal-transition, Prognosis, PI3K/AKT

## Abstract

**Background:**

Hepatocellular carcinoma (HCC) is one of the most prevalent malignancies worldwide because of rapid progression and high incidence of metastasis or recurrence. Accumulating evidence shows that CD73-expressing tumor cell is implicated in development of several types of cancer. However, the role of CD73 in HCC cell has not been systematically investigated and its underlying mechanism remains elusive.

**Methods:**

CD73 expression in HCC cell was determined by RT-PCR, Western blot, and immunohistochemistry staining. Clinical significance of CD73 was evaluated by Cox regression analysis. Cell counting kit-8 and colony formation assays were used for proliferation evaluation. Transwell assays were used for motility evaluations. Co-immunoprecipitation, cytosolic and plasma membrane fractionation separation, and ELISA were applied for evaluating membrane localization of P110β and its catalytic activity. NOD/SCID/γc(null) (NOG) mice model was used to investigate the in vivo functions of CD73.

**Results:**

In the present study, we demonstrate that CD73 was crucial for epithelial-mesenchymal transition (EMT), progression and metastasis in HCC. CD73 expression is increased in HCC cells and correlated with aggressive clinicopathological characteristics. Clinically, CD73 is identified as an independent poor prognostic indicator for both time to recurrence and overall survival. CD73 knockdown dramatically inhibits HCC cells proliferation, migration, invasion, and EMT in vitro and hinders tumor growth and metastasis in vivo. Opposite results could be observed when CD73 is overexpressed. Mechanistically, adenosine produced by CD73 binds to adenosine A2A receptor (A2AR) and activates Rap1, which recruits P110β to the plasma membrane and triggers PIP3 production, thereby promoting AKT phosphorylation in HCC cells. Notably, a combination of anti-CD73 and anti-A2AR achieves synergistic depression effects on HCC growth and metastasis than single agent alone.

**Conclusions:**

CD73 promotes progression and metastasis through activating PI3K/AKT signaling, indicating a novel prognostic biomarker for HCC. Our data demonstrate the importance of CD73 in HCC in addition to its immunosuppressive functions and revealed that co-targeting CD73 and A2AR strategy may be a promising novel therapeutic strategy for future HCC management.

**Electronic supplementary material:**

The online version of this article (10.1186/s13045-019-0724-7) contains supplementary material, which is available to authorized users.

## Introduction

Hepatocellular carcinoma (HCC) is one of the most prevalent malignancies worldwide and the second most common cause of cancer-related death [1, 2]. Although great improvements in treatment have been made, the prognosis of HCC remains unfavorable, with an approximate 30% overall 5-year survival rate even after curative resection [3–5]. Recurrence or metastasis is the major reason for the poor survival of HCC patients [6, 7], but the precise regulatory mechanisms of invasion and metastasis remain elusive. Therefore, identification of critical molecules that contribute to the invasive phenotype of HCC and clarification of the underlying mechanism are urgently needed to improve HCC prognosis.

CD73, an AMP hydrolyzing enzyme that regulates the conversion of extracellular ATP into adenosine, functions as a powerful immunosuppressor for maintaining tissue homeostasis and preventing immune responses during inflammation [8]. Accumulating evidence demonstrates the vital role of CD73 in tumor [9, 10]. Hematopoietic CD73 overexpression within the tumor microenvironment is observed in a variety of cancers, and these CD73-expressing cells hamper the immune reaction towards cancer cells and disable the cytotoxic antitumor immune response by producing high levels of adenosine [11–14]. CD73 expressed on hematopoietic cells can also function as a co-stimulatory molecule in human T cells to block CD8^+^ T cell activation [15]. Recent reports also demonstrated that CD73 overexpression is involved in migration, invasion, and angiogenesis [15–18]. Moreover, clinical data demonstrated that CD73 is a biomarker of poor prognosis in solid cancers [19–21]. These investigations suggested that CD73 could be a critical regulator that promotes tumor progression in an immune-independent manner. A preliminary study reported that CD73 was overexpressed in HCC cells and was positively correlated with EGFR expression [22]. However, whether CD73 could promote HCC progression and metastasis and the underlying regulatory mechanism still needs to be elucidated.

Here, we report that CD73 expression is positively correlated with metastasis in HCC and is an independent indicator for predicting prognosis. CD73 overexpression enhanced HCC progression and metastasis in the absence of an immunological environment in vitro and in vivo. Adenosine produced by CD73 binds to adenosine A2A receptor (A2AR) and activates Rap1, which recruits P110β to the plasma membrane and triggers PIP3 production, thereby promoting AKT phosphorylation. Importantly, the combination of CD73 and A2AR inhibitors provided more synergistic tumor inhibition than either regimen alone in HCC. 

## Methods and materials

### Clinical specimens and follow-up

Four groups of patients were recruited in the present study. In group I, frozen tumor tissues with paired paratumor normal tissues from 25 HCC patients receiving curative resection in October 2010 in Zhongshan Hospital were collected for comparisons of CD73 expression between matched tumor and paratumor tissues by RT-PCR and Western blot (WB) assays. In group II, eight recurrent HCC patients receiving curative resection from April to November 2014 in Zhongshan Hospital were enrolled, and matched frozen paratumor, primary tumor, and recurrent tumor tissues were collected for further WB assays. In group III, 10 HCC patients suffered lung metastasis after curative resection from 2011 to 2015 in Zhongshan hospital were enrolled, and matched paraffin sections of paratumor, primary tumor, and metastatic tumor tissues were collected for further immunohistochemical staining. In group IV, 189 HCC patients receiving curative resection in Zhongshan Hospital from March 2012 to September 2013 were enrolled, and paratumor and tumor specimens were collected for tissue microarrays (TMA) establishment. Enrollment criteria were according to a previous study [7]. HCC diagnosis was based on histopathology according to the American Association for Study of Liver Disease guidelines. The Barcelona Clinic Liver Cancer (BCLC) staging system was used to assess tumor stage [23]. Approval for the use of human subjects was obtained from the research ethics committee of Zhongshan Hospital, and informed consent was obtained from each individual. Follow-up ended on December 2016. Time to recurrence (TTR) was defined as the interval between treatment and intrahepatic recurrence or extrahepatic metastasis. Overall survival (OS) was defined as the interval between treatment and death of any cause or the last observation date.

### Statistical analysis

Statistical analyses were performed using SPSS 20.0 software (IBM, Chicago, IL, USA). Experimental values for continuous variables were expressed as the mean ± standard error of the mean. The chi-squared test, Fisher’s exact probability tests, and the Student’s *t* test were used as appropriate to evaluate the significance of differences in data between groups. If variances within groups were not homogeneous, a nonparametric Mann–Whitney test or a Wilcoxon signed-rank test was used. Prognostic value was evaluated by Kaplan–Meier survival curves, log-rank tests, and Cox proportional hazards models. A *P* value less than 0.05 was considered significant.

Further details of materials and methods are described in Additional file 1.

## Results

### CD73 is overexpressed in HCC tissues and correlates with poor prognosis

**Fig. 1:**
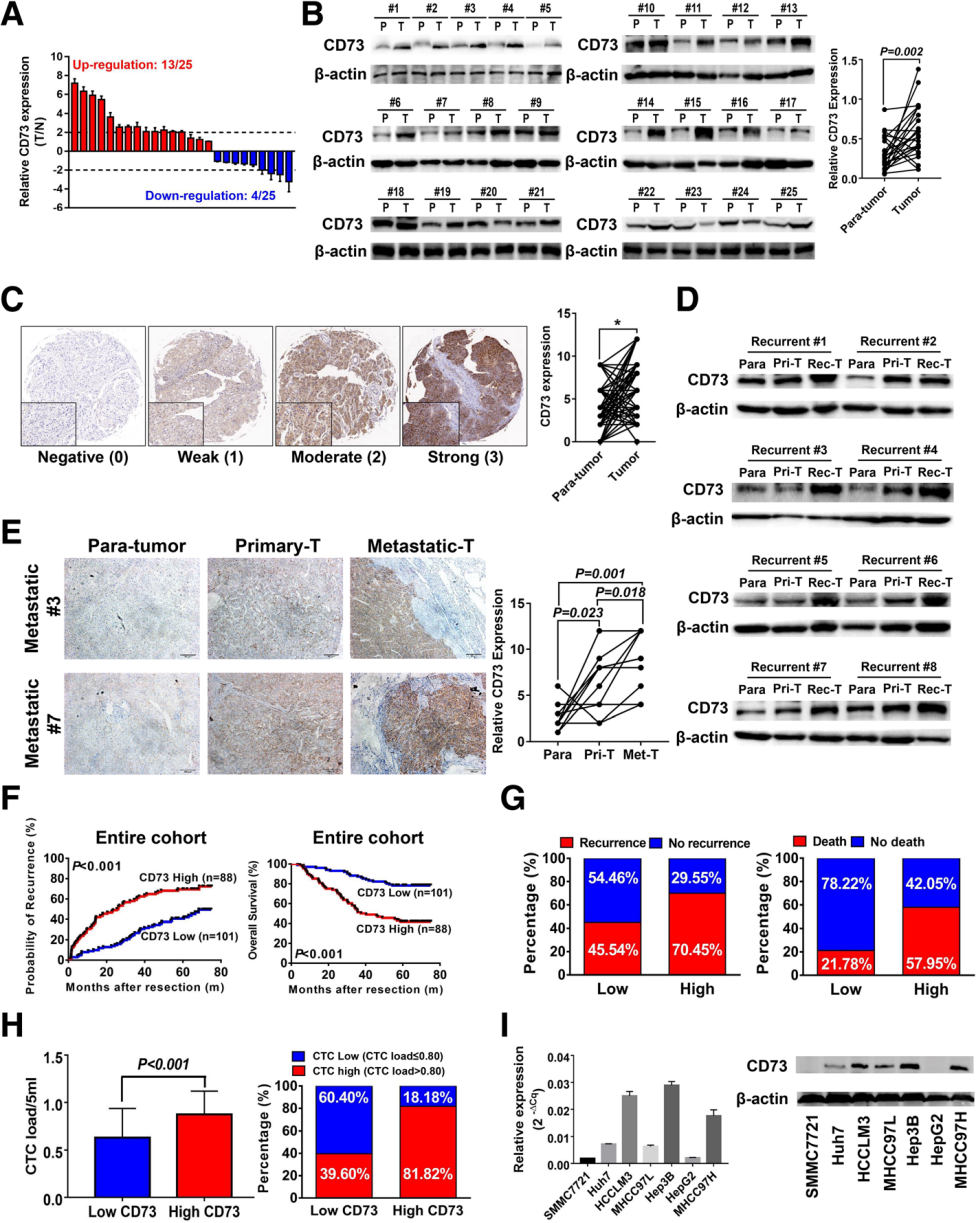
CD73 is overexpressed in HCC and indicates poor prognosis. **a** RT-PCR analysis of CD73 expression levels in 25 HCC tissues and paired non-tumor liver tissues. **b** Western blot (WB) analysis of CD73 expression levels in 25 HCC tissues and paired non-tumor liver tissues. *n* = 25; paired *t* test was used. **c** Representative immunohistochemistry (IHC) staining of CD73 expression, and comparison of the IHC score between cancerous and non-cancerous tissues (asterisk indicated *P* < 0.050, *n* = 189, non-parametric test was used). **d** WB analysis of CD73 expression levels in matched paratumor, primary-tumor, and recurrent tumor tissues from the same case (*n* = 8). **e** Representative IHC staining of CD73 expression in matched paratumor, primary-tumor, and metastatic tissues from the same case (left); comparisons of the IHC score were shown at the right panel (*n* = 10; non-parametric test was used). **f** Kaplan–Meier analysis of TTR (left) and overall survival (right) of HCC patients after curative resection according to CD73 expression level. **g** Recurrence (left) and death (right) incidences of HCC patients according to CD73 expression level. **h** Circulating tumor cell (CTC) load level of HCC patients according to CD73 expression level (left), and incidence of high CTC load in patients according to CD73 expression levels (right). (**i**) Expression level of CD73 in different HCC cell lines

CD73 expression was markedly higher in 55% of HCC tissues than paired adjacent normal liver tissues (Additional file 2: Figure S1A). WB assays confirmed the RT-PCR findings (Fig. 1b). Moreover, immunohistochemistry (IHC) staining was conducted with 189 HCC patients receiving curative resection, and results showed that HCC tissues expressed higher level of CD73 than adjacent non-cancerous liver tissues, according to the criteria of CD73 expression levels (Fig. 1c). These data revealed the potential oncogenic role of CD73 in HCC.

Next, the correlation between CD73 and HCC prognosis was evaluated. Samples from eight recurrent patients were collected. WB assays showed that expression levels of CD73 in matched non-tumor, primary tumor, and recurrence lesions exhibited an escalating pattern in most patients (Fig. 1d). For patients with metastasis, the CD73 expression level of metastasis foci was also significantly elevated according to IHC results (*n* = 10, Fig. 1e). Moreover, we found patients with high CD73 were prone to show incomplete tumor encapsulation, microvascular invasion, and poor differentiation (all *P* < 0.050, Table 1). Median TTR was significantly shorter in patients with high CD73 expression (median 25.73 months vs. not reached, *P* < 0.001, Fig. 1f), and the recurrence rate was also higher in these patients (70.45% vs. 45.54%, Fig. 1g). Similarly, patients with high CD73 expression had significantly shorter OS (median 38.88 months vs. not reached, *P* < 0.001) and a higher death rate (57.95% vs. 21.78%). Furthermore, CD73 retained its prognostic prediction value in early-stage and low-AFP subgroups (all *P* < 0.050, Additional file 2: Figure S1A–D). Notably, multivariate analysis demonstrated high CD73 expression on HCC cell and was an independent indicator for predicting both TTR [HR 2.82 (1.87–4.26), *P* < 0.001] and OS [HR 3.35 (2.01–5.52), *P* < 0.001, Tables 2 and 3].

**Table 1:** Correlation between clinicopathological parameters of patients enrolled

Clinical characteristics	No. of patients (*n* = 189)	CD73 low	CD73 high	*P*
Age, years				
≤ 50	79	35	44	**0.033**
> 50	110	66	44	
Sex				
Female	38	16	22	0.117
Male	151	85	66	
Child–Pugh score				
A	179	96	83	0.823^#^
B	10	5	5	
Liver cirrhosis				
No	42	20	22	0.391
Yes	147	81	66	
ALT, U/L				
≤ 40	134	71	63	0.845
> 40	55	30	25	
AST, U/L				
≤ 40	135	73	62	0.782
> 40	54	28	26	
AFP, ng/ml				
≤ 400	140	74	66	0.786
> 400	49	27	22	
No. of tumors				
Single	169	92	77	0.424
Multiple	20	9	11	
Tumor size, cm				
≤ 5	119	62	57	0.631
> 5	70	39	31	
Tumor encapsulation				
Complete	123	59	64	**0.040**
None	66	42	24	
Satellite lesion				
No	171	88	83	0.093
Yes	18	13	5	
Macrovascular invasion				
No	175	94	81	0.784
Yes	14	7	7	
Microvascular invasion				
No	107	64	43	**0.045**
Yes	82	37	45	
Edmondson stage				
I-II	126	74	52	**0.039**
III-IV	63	27	36	
BCLC stage				
0 + A	158	86	72	0.537
B + C	31	15	16	

*ALT* alanine aminotransferase, *AST* aspartate transaminase, *AFP* α-fetoprotein, *BCLC* Barcelona Clinic Liver Cancer

Bold indicated statistical significance

**Table 2:** Univariate Cox proportional regression analysis of factors associated with recurrence and overall survival

Variables	Recurrence		Overall survival	
	HR (95% CI)	*P*	HR (95% CI)	*P*
Age (> 50 years versus ≤ 50 years)	0.73 (0.50–1.07)	0.104	0.64 (0.41–1.02)	0.060
Sex (male versus female)	0.76 (0.48–1.21)	0.249	1.16 (0.69–1.95)	0.563
Liver cirrhosis (yes versus no)	1.52 (0.92–2.49)	0.099	1.11 (0.63–1.96)	0.712
ALT (> 40 U/L versus ≤ 40 U/L)	1.57 (1.06–2.34)	**0.025**	1.8 (0.85–2.24)	0.190
AST (> 40 U/L versus ≤40 U/L)	1.78 (1.20–2.64)	**0.004**	1.49 (0.92–2.41)	0.101
AFP (> 400 ng/ml versus ≤ 400 ng/ml)	1.86 (1.25–2.78)	**0.002**	1.49 (0.91–2.43)	0.117
No. of tumors (multi versus single)	1.81 (1.06–3.08)	**0.030**	1.57 (0.84–3.02)	0.157
Tumor size (> 5 cm versus ≤ 5 cm)	2.35 (1.60–3.43)	**< 0.001**	1.59 (0.99–2.48)	0.056
Tumor encapsulation (none versus complete)	1.15 (0.78–1.69)	0.452	1.03 (0.64–1.67)	0.903
Satellite lesions (yes versus no)	1.67 (0.96–2.88)	0.069	0.78 (0.33–1.76)	0.528
Macrovascular invasion (yes versus no)	2.30 (1.28–4.12)	**0.005**	2.19 (1.13–4.28)	**0.021**
Microvascular invasion (yes versus no)	2.08 (1.42–3.04)	**< 0.001**	1.91 (1.21–3.04)	**0.006**
Edmondson stage (III–IV versus I–II)	1.78 (1.21–2.61)	**0.003**	1.50 (0.94–2.39)	0.087
BCLC stage (B + C versus 0 + A)	1.98 (1.27–3.09)	**0.003**	1.72 (1.01–2.94)	**0.045**
ALBI grade (II versus I)	1.23 (0.80–1.89)	0.344	1.86 (1.12–3.09)	**0.017**
CD73 (high versus low)	2.33 (1.59–3.42)	**< 0.001**	3.64 (2.20–6.01)	**< 0.001**

Bold indicated statistical significance

**Table 3:** Multivariate cox proportional regression analysis of factors associated with recurrence and overall survival

Variables	Recurrence		Overall survival	
	HR (95% CI)	*P*	HR (95% CI)	*P*
ALT (> 40 U/L versus ≤ 40 U/L)	1.24 (0.78–1.96)	0.369	N.A.	N.A.
AST (> 40 U/L versus ≤ 40 U/L)	1.46 (0.93–2.27)	0.099	N.A.	N.A.
AFP (> 400 ng/ml versus ≤ 400 ng/ml)	1.94 (1.29–2.94)	**0.002**	N.A.	N.A.
No. of tumors (multi versus single)	4.71 (1.28–17.42)	**0.020**	N.A.	N.A.
Tumor size (> 5 cm versus ≤ 5 cm)	2.01 (1.29–3.13)	**0.002**	N.A.	N.A.
Macrovascular invasion (yes versus no)	3.19 (0.83–12.29)	0.092	1.59 (0.95–2.67)	0.078
Microvascular invasion (yes versus no)	1.18 (0.75–1.85)	0.471	1.86 (1.16–2.96)	**0.009**
Edmondson stage (III–IV versus I–II)	1.33 (0.88–2.02)	0.182	0.98 (0.60–1.61)	0.932
ALBI grade (II versus I)	1.51 (0.63–3.60)	0.355	1.89 (1.09–3.18)	**0.021**
CD73 (high versus low)	2.82 (1.87–4.26)	**< 0.001**	3.35 (2.01–5.52)	**< 0.001**

Bold indicated statistical significance

Intriguingly, patients with high CD73 had significantly higher pre-treatment circulating tumor cell (CTC) load (*P* < 0.001, Fig. 1h). Using 0.80 as the cutoff value for CTC load [24], a higher proportion of patients in the CD73^high^ group had high CTC loads (Fig. 1h). Notably, CD73 showed elevated expression in MHCC97H and HCCLM3 cells, which have higher invasive and metastatic potentials (Fig. 1i). In comparison, cell lines with lower invasive and metastatic capacities such as SMMC7721 and MHCC97L showed relatively low CD73 levels. Collectively, our data demonstrated the clinical significance of CD73 and suggested it as a potential promoter for HCC metastasis/recurrence.

### CD73 promotes HCC progression in vitro

**Fig. 2:**
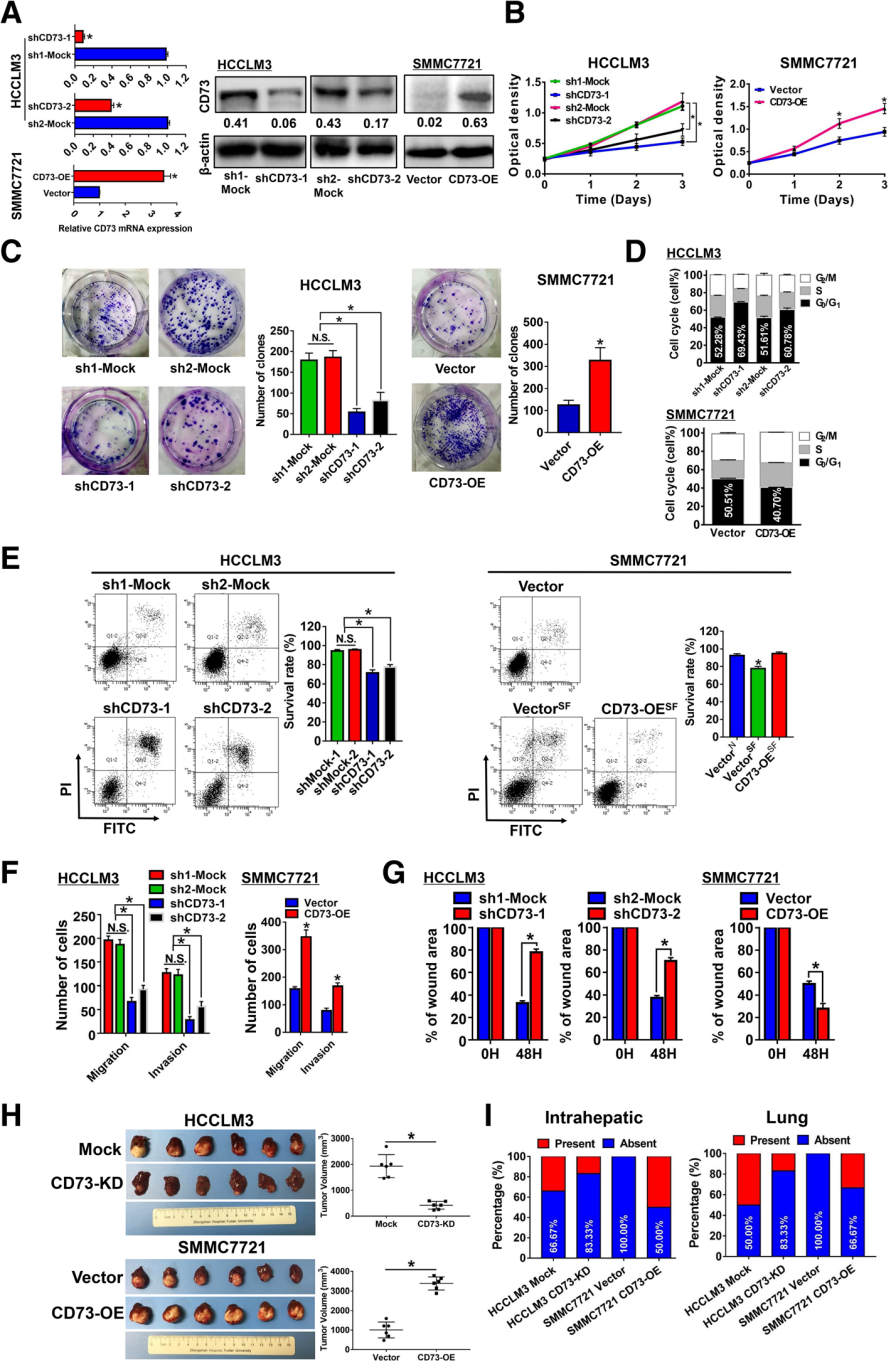
Biological functions of CD73 in HCC. **a** Efficiencies of CD73 knockdown and overexpression were validated by RT-PCR (left) and WB (right) assays. **b** Evaluation of the influence of CD73 on HCC proliferation by CCK-8. **c** Evaluations of the influence of CD73 on HCC proliferation by colony formation assays. **d** Evaluations of the influence of CD73 on cell cycle in HCC cells by flow cytometry. **e** Evaluations of the influence of CD73 on apoptosis in HCC cells by flow cytometry. **f** Evaluations of the influence of CD73 on migration and invasion activities of HCC cells by Transwell assays. **g** Evaluations of the influence of CD73 on migration activities of HCC cells by wound healing assays. **h** Establishment of the orthotopic xenograft model with NOG mice. Tumor volumes are shown in the right panel. **i** Incidences of intrahepatic (left) or lung (right) metastasis in indicated orthotopic xenograft model groups. All in vitro experiments were performed in triplicate; asterisk indicated *P* < 0.050, and *t* tests were used

CD73 expression in HCC cell lines was manipulated by ectopic expression or short hairpin RNA (shRNA) knockdown. Two distinct shRNAs were designed to knock down CD73 expression in two CD73^high^ HCC cell lines, HCCLM3 and Hep3B cells. Knockdown effects were validated by RT-PCR and WB assays (Fig. 2a, Additional file 3: Figure S2A). Stable ectopic expression of CD73 in a CD73^low^ HCC cell line, SMMC7721, was also confirmed by RT-PCR and WB assays. We found CD73^KD^ HCCLM3 cells exhibited significantly decreased proliferation potential compared with controls according to CCK-8 and colony formation assays (Fig. 2b and c). Similar reduced proliferation was observed in Hep3B cells (Additional file 3: Figure S2B). In contrast, overexpression of CD73 in SMMC7721 cells increased their proliferation in CCK-8 assays and clonogenicity capacity (Fig. 2b and c). Cell cycle assays demonstrated that CD73 knockdown resulted in G0/G1 arrest in HCC cells (Fig. 2d, Additional file 3: Figure S2C), while CD73 overexpression accelerated the cell cycle in SMMC7721 cells (Fig. 2d). Apoptosis assays further showed that CD73 knockdown greatly induced apoptosis in HCC cells, while CD73 overexpression protected SMMC7721 cells from a serum-free environment (Fig. 2e, Additional file 3: Figure S2D). Transwell assays showed that CD73 knockdown HCC cells had significantly fewer migrating and invading cells than parental cells, while CD73^OE^ SMMC7721 significantly higher migratory and invasive capacities (Fig. 2f, Additional file 4: Figure S3A and B). Moreover, wound healing assays confirmed the findings of Transwell assays that knockdown of CD73 greatly hindered HCC migration, while overexpression of CD73 promoted migratory potentials (Fig. 2g, Additional file 4: Figure S3C). Importantly, we also conducted CD73 overexpression in HCCLM3 cells to confirm that the difference observed above was not due to the variability between cell lines. CD73 expression was greatly increased after plasmid transfection in HCCLM3 cells (Additional file 5: Figure S4A). Similar to the effects of CD73 overexpression on SMMC7721 cells, forced expression of CD73 also significantly promoted proliferation, migration, and invasion capacities in HCCLM3 cells (Additional file 5: Figure S4B–D). However, no significant alteration of apoptosis and cell cycle was observed after CD73 overexpression (Additional file 5: Figure S4E and F). Taken together, our data indicated that CD73 promotes HCC cell proliferation, migration, and invasion capacities and prevents apoptosis in vitro.

### CD73 promotes HCC growth and metastasis in vivo

Since shCD73-1 exhibited more efficiency in knocking down CD73 expression and resulting in more significant biological alterations, further in vivo experiments and mechanism investigation were conducted using this shRNA. To minimize the impact of the immune system, NOG mice were used. Model mice were sacrificed after 6 weeks, and analyses of liver orthotopic xenograft tumors showed that CD73 knockdown greatly inhibited tumor growth, whereas CD73 overexpression promoted tumor growth in vivo (Fig. 2h). Moreover, tumor tissues with high CD73 expression also exhibited high proliferating cell nuclear antigen levels (Additional file 6: Figure S5A). Metastatic foci were examined based on their tissue structure and cell morphology by two senior histopathologists to evaluate the effects of CD73 on HCC metastasis. Both the intrahepatic and pulmonary metastasis rates in mice with tumors generated from CD73^KD^ HCCLM3 cells were lower than in mice with tumors derived from parental HCCLM3 cells (Fig. 2i, Additional file 6: Figure S5B and C). In contrast, both intrahepatic and pulmonary metastasis rates were increased in tumors derived from CD73^OE^ SMMC7721 cells compared with parental SMMC7721 cells. Collectively, our data demonstrated that CD73 promotes HCC proliferation and metastasis in vivo.

### CD73 triggers epithelial-mesenchymal transition in HCC

**Fig. 3:**
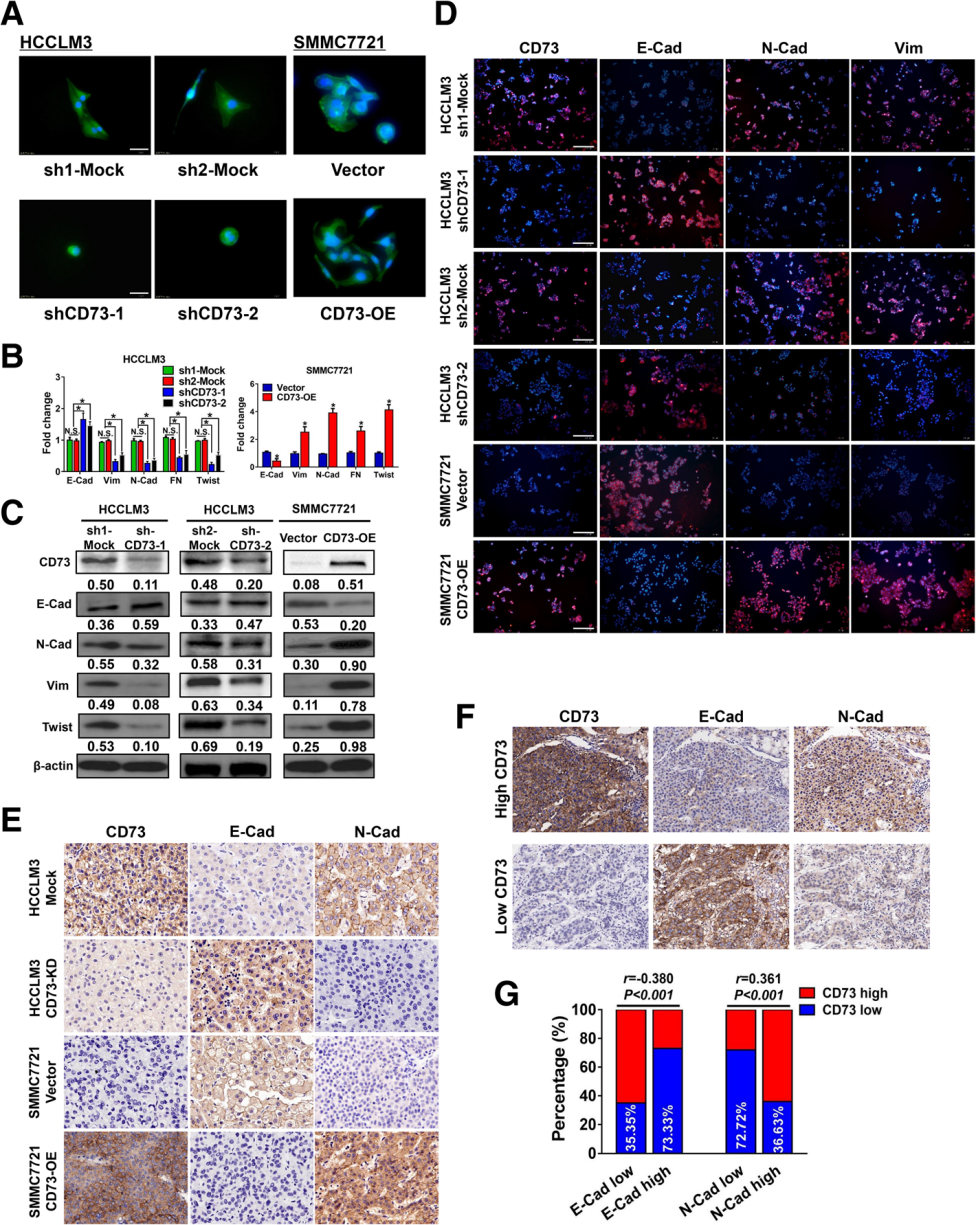
CD73 triggers EMT in HCC. **a** Representative images of HCC cells with CD73 downregulation or overexpression. Cells were stained by FITC-phalloidin; scale bar 20 μm. **b** Expressions of EMT-related markers in HCC cells with CD73 downregulation or overexpression by RT-PCR assays. **c** Expressions of EMT-related markers in HCC cells with CD73 downregulation or overexpression by WB assays. **d** Expressions of CD73, E-Cadherin, N-Cadherin, and Vimentin in HCC cell lines with CD73 downregulation or overexpression by immunofluorescence staining; scale bar 200 μm. **e** Representative IHC images of CD73, E-Cadherin, and N-Cadherin expressions in tumor tissues derived from HCC cell lines as indicated. **f** Representative IHC images of CD73, E-Cadherin, and N-Cadherin expressions in consecutive tissue sections of clinical HCC samples. **g** Correlations between CD73 and E-Cadherin or N-Cadherin in clinical HCC samples were analyzed by Spearman’s rank correlation test. PCR assays were performed in triplicate. “N.S.” indicated not significant; asterisk indicated *P* < 0.050, *t* tests were used

We further examined whether CD73 contributes to EMT. Through phalloidin staining, we found CD73^KD^ HCCLM3 cells showed an epithelial cobblestone phenotype with less pseudopod, whereas CD73^OE^ SMMC7721 cells transformed into a spindle-like shape with more pseudopods (Fig. 3a). Moreover, CD73 knockdown resulted in an epithelial-like molecular phenotype, while CD73 overexpression induced a mesenchymal-like molecular phenotype according to RT-PCR and WB results (Fig. 3b and c). Consistently, immunofluorescence analysis confirmed these findings (Fig. 3d). Moreover, xenograft tumors generated from CD73^KD^ HCCLM3 cells exhibited an epithelial-like phenotype, while tumors derived from CD73^OE^ SMMC7721 cells showed a mesenchymal-like phenotype according to IHC staining (Fig. 3e). We next examined the correlation between CD73 and EMT in a clinical cohort. IHC staining in HCC consecutive sections indicated that high CD73 expression was correlated with low level of E-Cadherin but high level of N-Cadherin, and vice versa in HCC tissue with low CD73 expression (Fig. 3f). Notably, correlation analysis revealed that high CD73 expression was positively correlated with N-Cadherin but negatively correlated with E-Cadherin expression in clinical HCC samples (Fig. 3g). Collectively, our results suggested that CD73 is a critical regulator of EMT in HCC.

### CD73 promotes HCC progression and EMT by activating the PI3K-AKT signaling pathway

**Fig. 4:**
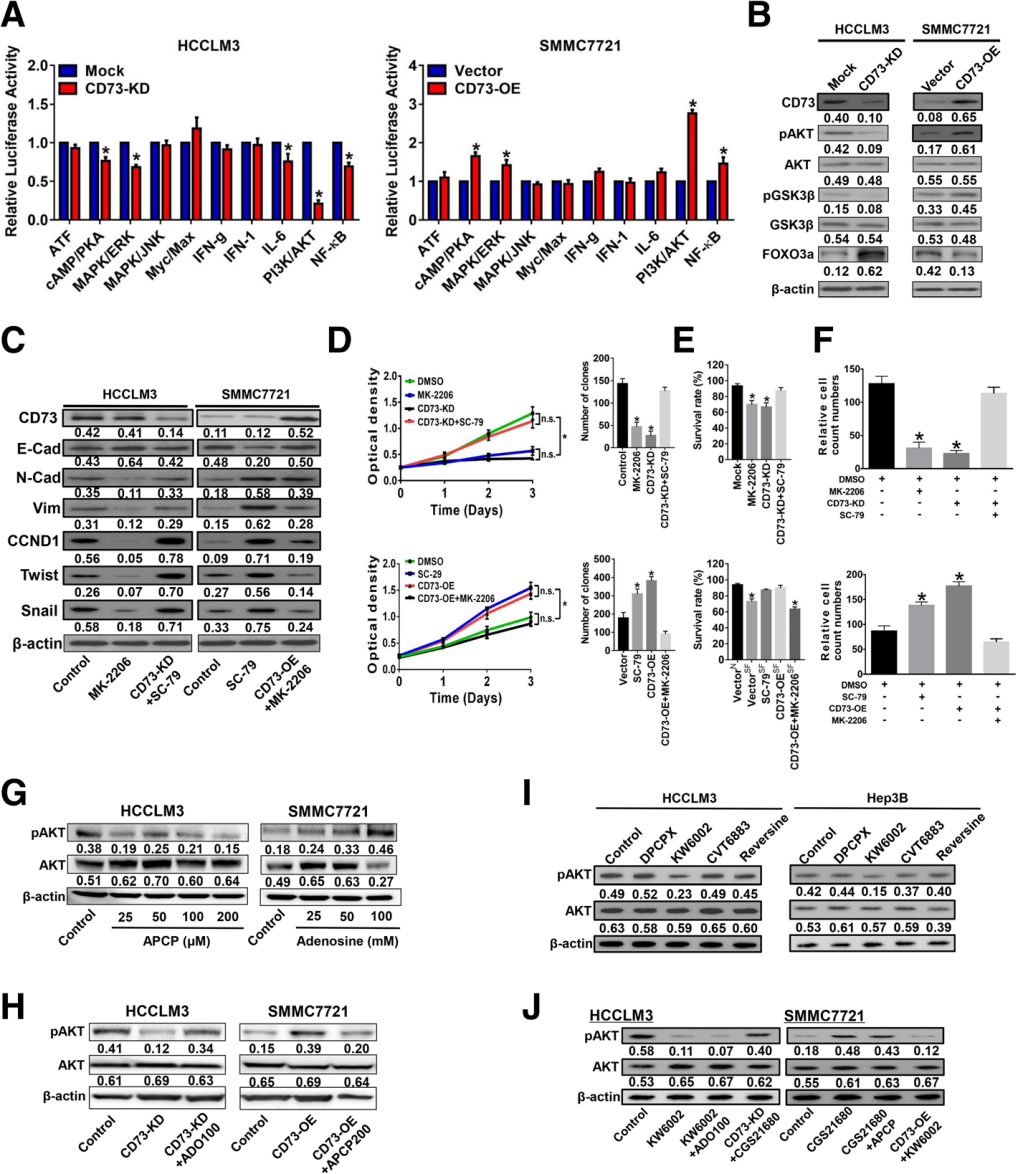
CD73 require its enzymatic activity and occur through adenosine receptor A2A to activate the PI3K/AKT pathway. **a** Cignal Finder RTK signaling 10-Pathway Reporter Array results demonstrate signaling changes in the indicated HCC cells. **b** Phosphorylation levels of AKT, GSK3β, and FOXO3a expression levels in the indicated HCC cells as determined by WB assays. **c** Expressions of EMT-related markers in the indicated HCC cells were detected by WB assays. **d** Proliferation in the indicated HCC cell lines was evaluated by CCK-8 and colony formation assays. “N.S.” indicated not significant; asterisk indicated *P* < 0.050, and *t* tests were used. **e** Apoptosis in the indicated HCC cell lines was evaluated by flow cytometry assays; asterisk indicated *P* < 0.050 when compared with control group, and *t* tests were used. **f** Invasion in the indicated HCC cell lines was evaluated by Transwell assays; asterisk indicated *P* < 0.050 when compared with control group, and *t* tests were used. **g** Phosphorylation levels of AKT in HCCLM3 cells under different concentrations of APCP (left) or in SMMC7721 cells under different concentrations of adenosine treatments (right) as detected by WB assays. **h** Phosphorylation levels of AKT in the indicated HCCLM3 (left) or SMMC7721 cells (right) as detected by WB assays. **i** Phosphorylation levels of AKT in HCCLM3 (left) or Hep3B cells (right) treated with antagonists targeting specific adenosine receptors (A1R, DPCPX; A2AR, KW6002; A2BR, CVT6883; A3R, Reversine). **j** Phosphorylation levels of AKT in indicated HCCLM3 (left) or SMMC7721 cells (right) as detected by WB assays. All in vitro experiments were performed in triplicate

To identify the underlying signaling of CD73 in HCC, Cignal Finder RTK signaling 10-Pathway Reporter Array was used and results showed that PI3K/AKT signaling exhibited the greatest fold changes due to CD73 expression manipulation (Fig. 4a). We therefore examined the PI3K/AKT pathway as a candidate critical signaling pathway. WB assays in HCC cells with CD73 knockdown confirmed that phosphorylation levels of AKT and GSK3β, a direct substrate of pAKT, were reduced (Fig. 4b). In addition, expression of FOXO3a, which would undergo degradation due to AKT activation, was also increased after CD73 knockdown. In contrast, pAKT and pGSK3β levels were and increased, while FOXO3a was decreased with ectopic expression of CD73 in SMMC7721 cells (Fig. 4b). We next examined whether the effects of CD73 on HCC cell activities were dependent on PI3K/AKT signaling. Treatment of HCCLM3 cells with MK-2206, an AKT inhibitor, resulted in an epithelial-like molecular phenotype, which resembled the phenotype of CD73^KD^ HCCLM3 cells (Fig. 4c, Additional file 7: Figure S6A). Treatment of SMMC7721 cells with SC-79, an AKT activator, resulted in a mesenchymal-like phenotype that resembled the phenotype of CD73^OE^ SMMC7721 cells. Importantly, reactivation of AKT signaling in CD73^KD^ HCCLM3 cells effectively attenuated the epithelial-induction effect, while inhibition of AKT signaling in CD73^OE^ SMMC7721 cells prevented the mesenchymal-induction effect (Fig. 4c). Also, biological experiments showed that AKT inhibition hindered proliferation and invasion capacities and promoted apoptosis in CD73^high^ HCCLM3 cells to levels observed in CD73 knockdown cells, while AKT activation promoted proliferation and invasion capacities and prevented apoptosis in CD73^low^ SMMC7721 cells to levels observed in cells with CD73 overexpression (Fig. 4d–f, Additional file 7: Figure S6B–D). Notably, AKT inhibition abolished the effects of CD73 overexpression on HCC proliferation and invasion, whereas AKT activation attenuated the inhibitory effects of CD73 knockdown on HCC proliferation and invasion. These results indicated that CD73 promotes HCC progression and EMT by activating PI3K/AKT signaling.

### CD73 function in HCC mainly depends on its enzymatic activity and occurs through adenosine receptor A2A

We next investigated whether the enzymatic activity of CD73 was required for its function in promoting progression and metastasis in HCC by using APCP, a CD73 enzyme activity inhibitor. APCP treatment of CD73^high^ HCCLM3 cells reduced pAKT levels in a dose-dependent manner (Fig. 4g). Contrarily, exogenous adenosine treatment of CD73^low^ SMMC7721 cells increased pAKT in a dose-dependent manner. Notably, adenosine could restore the reduction of pAKT caused by CD73 knockdown, while APCP treatment could partly abolish the increase of pAKT caused by CD73 overexpression (Fig. 4h). Functionally, APCP produced similar inhibitory effects on proliferation and invasion, induced an epithelial-like phenotype in HCCLM3 cells as observed in CD73 knockdown cells, and abolished the effects of CD73 overexpression (Additional file 8: Figure S7A-C), whereas adenosine promoted proliferation and invasion, induced a mesenchymal-like phenotype in SMMC7721 cells as observed in CD73 overexpression cells, and attenuated the effects of CD73 knockdown (Additional file 8: Figure S7D–F). Collectively, our data demonstrated that CD73 functions in HCC require its enzymatic activity.

We next searched for the adenosine receptor involved in mediating the functions of CD73 in HCC using selective inhibitors targeting adenosine A1 (DPCPX), A2A (KW6002), A2B (CVT6883), and A3 receptors (Reversine). Only KW6002 reduced AKT phosphorylation in both CD73^high^ HCC cell lines (Fig. 4i). Furthermore, KW6002 inhibited HCC proliferation and invasion to the maximum extent and induced an epithelial-like phenotype (Additional file 9: Figure S8A–F). We thus focused on A2A receptor (A2AR) for further investigation. Inhibition effects of A2AR on pAKT level could not be rescued by exogenous adenosine, while APCP failed to abolish the increase of pAKT resulting from using CGS21650, a selective A2AR agonist (Fig. 4j). Importantly, A2AR agonist could effectively restore the reduction of pAKT caused by CD73 knockdown, while A2AR antagonist greatly abolished the increase of pAKT resulting from CD73 overexpression (Fig. 4j). These data demonstrated that CD73 exerts its function in HCC through A2AR.

### The CD73-A2AR axis induces Rap1 activation and promotes its membrane recruitment

**Fig. 5:**
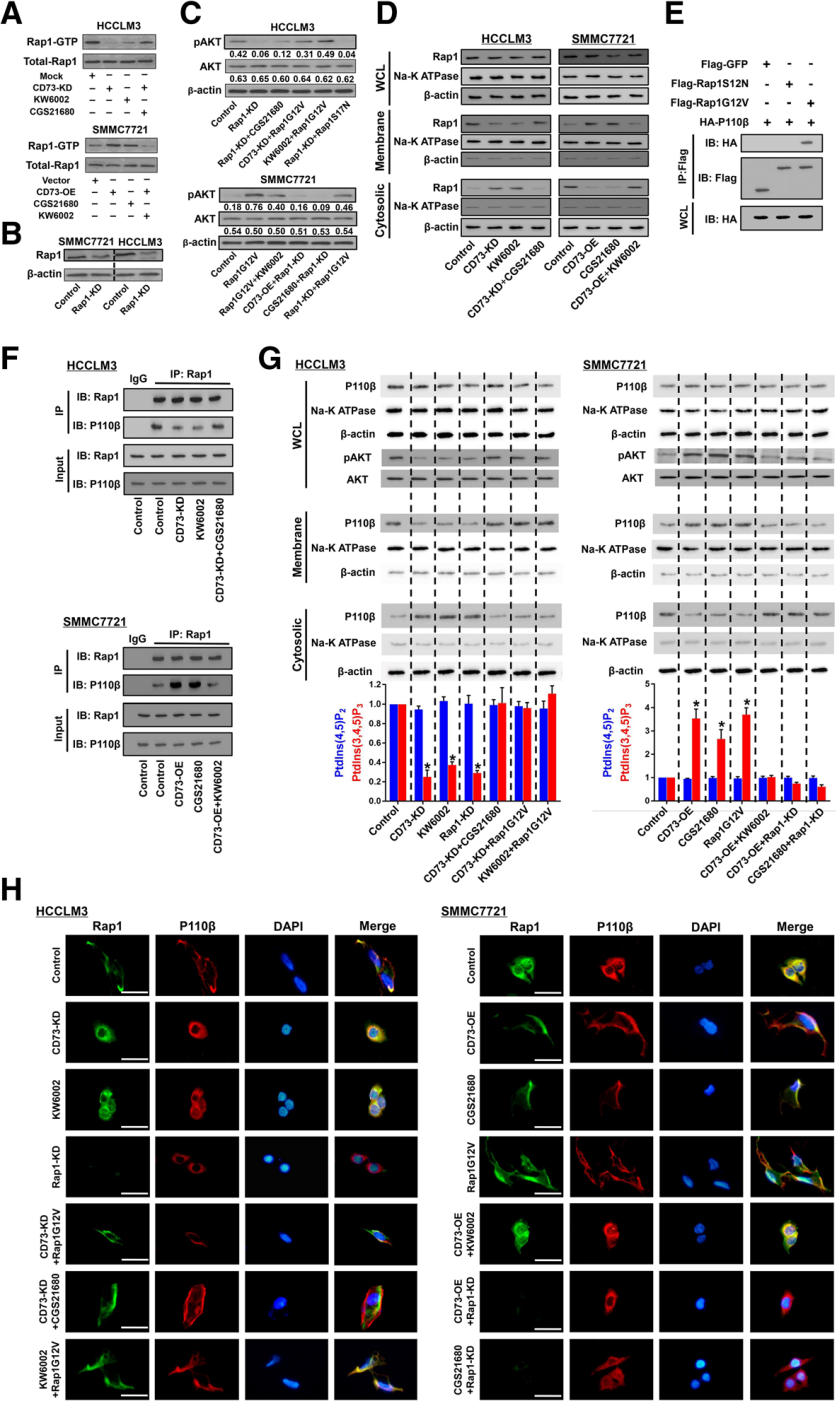
CD73-A2AR axis activates PI3K/AKT signaling by inducing Rap1-mediated membrane localization of P110β. **a** Rap1 activation status was detected in the indicated HCC cells. **b** Rap1 expression was silenced via using shRNA in HCCLM3 and SMMC7721 cells and was confirmed by WB assays. **c** pAKT levels in the indicated HCCLM3 (upper) or SMMC7721 cells (lower) by WB assays. **d** Cytosolic and plasma membrane fractions were isolated from the indicated HCCLM3 (left) or SMMC7721 cells (right), followed by WB assays with antibody against Rap1. Na^+^-K^+^ ATPase was used as an internal control for membrane fractions, and β-actin was used as an internal control for cytosolic fractions. **e** Co-immunoprecipitation of HA-P110β and Flag-Rap1G12V or Flag-Rap1S12N in SMMC7721 cells. **f** Effects of the CD73-A2AR axis on promoting Rap1-P110β interaction in co-IP assays. **g** Cytosolic and plasma membrane fractions were isolated from the indicated HCCLM3 (left) or SMMC7721 cells (right), followed by WB assays with antibody against P110β. pAKT level and PIP3 concentrations were also detected. ELISA experiments for PIP3 determination were conducted in triplicate. **h** Immunofluorescence staining of Rap1 (green) and P110β (red) with specific antibodies in indicated HCCLM3 (left) or SMMC7721 cells (right). DAPI was used for nuclear staining; scale bar 50 μm. Asterisk indicated *P* < 0.050 when compared with control group, *t* tests were used

Adenosine A2AR belongs to the G-protein-coupled receptor family, which is closely associated with the activation of Rap1 [25]. Previous studies showed that Rap1 is a critical regulator of PI3K activity [26, 27]. We therefore hypothesized that the CD73-A2AR axis might activate PI3K/AKT signaling by inducing Rap1 activation. We first assessed the effects of the CD73-A2AR axis on Rap1 activation. CD73 knockdown or A2AR antagonist greatly reduced the level of Rap1-GTP, an active form of Rap1, while A2AR agonist restored the reduction of Rap1-GTP level caused by CD73 knockdown in HCCLM3 cells (Fig. 5a). Conversely, CD73 overexpression or A2AR agonist increased Rap1-GTP, while A2AR antagonist abolished the increase of Rap1-GTP level resulting from CD73 overexpression.

We next determined the role of Rap1 activation in AKT phosphorylation via manipulating Rap1 expression (Fig. 5b). In HCCLM3 cells, Rap1 knockdown dramatically decreased pAKT levels, which could not be rescued by CGS21680 treatment (Fig. 5c). However, transfection of Rap1G12V, a constitutively active mutant, attenuated the reduction of pAKT level caused by CD73 knockdown or A2AR antagonist. Moreover, transfection of Rap1S17N, a dominant negative mutant, failed to restore the inhibitory effects of Rap1 knockdown on pAKT levels (Fig. 5c). In SMMC7721 cells, transfection of Rap1G12V greatly increased pAKT levels, which could not be abolished by A2AR antagonist. However, CD73 overexpression or A2AR agonist showed no effects on pAKT level when Rap1 was knocked down. In addition, Rap1G12V expression rescued the inhibition of AKT activation caused by Rap1 knockdown (Fig. 5c). Importantly, through analyzing cytosolic and plasma membrane fractions, we found that the majority of Rap1 was recruited to the membrane after activation by the CD73-A2AR axis (Fig. 5d), which was consistent with a previous study [28]. These results indicate that Rap1 serves as a key downstream regulator of the CD73-A2AR axis and that activation of Rap1 is essential for CD73-A2AR axis mediating AKT phosphorylation in HCC.

### The CD73-A2AR axis promotes Rap1-P110β interaction

Previous studies reported that Rap1 could bind to Class I PI3Ks to promote PIP3 production, resulting in AKT activation [26, 27]. Thus we hypothesized that CD73-A2AR axis activates AKT signaling via promoting interaction between Class I PI3K member and Rap1. First, to identify the key involved Class I PI3K member, we silenced two major Class I PI3Ks, P110α, and P110β, in HCCLM3 cells and found that P110β knockdown resulted in a dramatic reduction of pAKT levels (Additional file 10: Figure S9A). Similar results were observed in Hep3B cells (Additional file 10: Figure S9B). These results suggested a critical role for P110β in CD73-mediated AKT activation in HCC. Next, Co-IP experiments in 293 T cells co-transfected with HA-P110β and either Flag-Rap1G12V or Flag-Rap1S12N showed that only activated form Rap1 could interact with P110β (Fig. 5e). Notably, level of intracellular PIP3 and pAKT only elevated when Rap1 binding to P110β, implying a requirement for this interaction in promoting catalytic activity of P110β (Additional file 10: Figure S9C, D).

Above data demonstrated the significance of the interaction between Rap1 and P110β in regulating AKT activation. Next, the role of CD73-A2AR axis in promoting endogenous Rap1-P110β interaction was further validated by co-IP assays. CD73 knockdown or A2AR antagonist greatly hindered the Rap1-P110β interaction, whereas A2AR agonist successfully rescued the inhibition of Rap1-P110β interaction caused by CD73 knockdown in HCCLM3 cells (Fig. 5f). Contrarily, CD73 overexpression or A2AR agonist promoted the Rap1-P110β interaction, while A2AR antagonist dramatically inhibited the effects of CD73 overexpression on promoting Rap1-P110β binding in SMMC7721 cells. Our findings indicate that CD73-A2AR serves as an upstream promoter of the Rap1-P110β interaction, resulting in PIP3 production and AKT phosphorylation.

### The CD73-A2AR axis promotes Rap1-mediated membrane localization of P110β and PIP3 production

As our results showed that activated Rap1 was recruited to the membrane, and Rap1 binds to P110β, we further evaluated whether CD73-A2AR could induce P110β membrane localization, a critical process for its catalytic function [29], in a Rap1-dependent manner. Through analyzing cytosolic and plasma membrane fractions by WB assays, we found that, similar to the effect of Rap1 knockdown, CD73 knockdown or A2AR antagonist greatly reduced the plasma membrane localization of P110β in HCCLM3 cells, and this could be rescued by Rap1G12V expression (Fig. 5g). On the other hand, consistent with the results of Rap1G12V transfection, CD73 overexpression or A2AR agonist promoted plasma membrane localization of P110β in SMMC7721 cells, and this was greatly abolished by Rap1 knockdown. Notably, higher PIP3 concentrations and pAKT levels were observed when the majority of P110β localized on plasma membrane (Fig. 5g), indicating abundant catalytic activity of P110β under these circumstances. Assays were further performed and results further confirmed the membranous co-localization of Rap1-P110β due to CD73-A2AR axis regulation, which was consistent with the results of WB assays (Fig. 5h). These data demonstrated that the CD73-A2AR axis activates AKT by inducing Rap1-mediated membrane localization of P110β.

### CD73 is negatively regulated by miR-193b

**Fig. 6:**
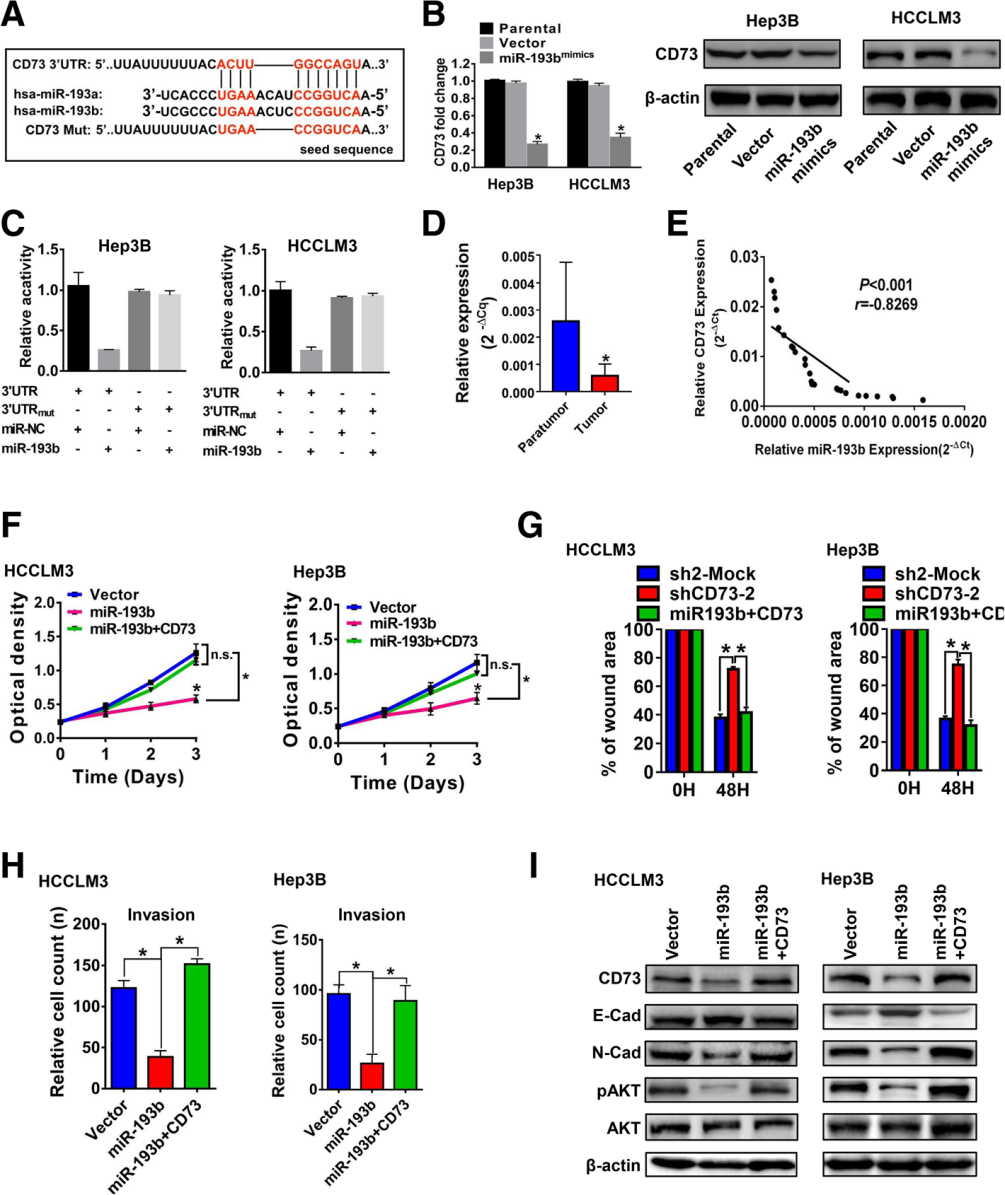
CD73 is regulated by miR-193b. **a** Putative binding site of miR-193a/miR-193b and CD73. **b** Expression of CD73 in Hep3B and HCCLM3 cells after miR-193b mimic transfection as detected by RT-PCR and WB assays; asterisk indicated *P* < 0.050 when compared with parental group, and *t* tests were used. **c** Effect of miR-193b on the activity of CD73 mRNA 3′UTR was evaluated by luciferase reporter assays. **d** Comparison of miR-193b expression level between paratumor and tumor tissues determined by RT-PCR assays; asterisk indicated *P* < 0.050, *n* = 25, and *t* tests were used. **e** Correlation of miR-193b and CD73 mRNA in clinical HCC samples was analyzed by Spearman’s rank correlation test, *n* = 25. **f** Evaluations of proliferation capacities of indicated HCCLM3 (left) and Hep3B cells (right) by CCK-8 assays; “N.S.” indicated not significant; asterisk indicated *P* < 0.050, and *t* tests were used. **g** Evaluations of migration capacities of indicated HCCLM3 (left) and Hep3B cells (right) by wound healing assays; asterisk indicated *P* < 0.050, and *t* tests were used. **h** Evaluations of invasion capacities of indicated HCCLM3 (left) and Hep3B cells (right) by wound healing assays; asterisk indicated *P* < 0.050, *t* tests were used. **i** Evaluations of EMT-related genes and pAKT expression levels of indicated HCCLM3 (left) and Hep3B cells (right) by WB assays. In vitro experiments were performed in triplicate

Previous studies revealed miRNA was responsible for the dysregulation of CD73 expression [30, 31]. We further investigated the potential upstream miRNA of CD73 in HCC. Seven microRNAs were predicted by two bioinformatics algorithms (TargetScan and miRanda) to be potential upstream regulators of CD73 according to StarBase database [32] (Additional file 11: Figure S10A). Among these potential miRNAs, two homologous miRNAs, miR-193a and miR-193b were selected for further study, as previous studies reported them as tumor suppressors in HCC [33, 34]. A putative binding site of miR-193a/miR-193b and 3′UTR of CD73 was identified (Fig. 6a). Transfection of miR-193b mimics significantly reduced CD73 expression in two CD73^high^ cell lines (Fig. 6b). However, miR-193a mimics failed to exert similar effects (Additional file 11: Figure S10B), and thus, miR-193b was identified as the potential regulator for CD73 expression in our study. To validate its regulatory role, dual luciferase reporter assays were firstly performed and results revealed that miR-193b markedly inhibited the activity of a luciferase vector containing the wild-type CD73 3′UTR, but not the mutant 3′UTR (Fig. 6c). Moreover, miR-193b expression was significantly downregulated in HCC tissues compared to paratumor tissues (Fig. 6d) and reversely correlated with CD73 mRNA in clinical HCC samples (*r* = − 0.83, *P* < 0.001, Fig. 6e). Functional assays demonstrated that transfection of miR-193b could significantly inhibit HCC proliferation, migration, and invasion, and these inhibitory effects could be rescued by CD73 re-expression (Fig. 6f–h, Additional file 11: Figure S10C and D). Additionally, induction of epithelial-like phenotype and downregulation of phosphorylation of AKT caused by miR-193b mimic transfection could also be reversed by re-expression of CD73 (Fig. 6i). Taken together, our data identified miR-193b as a novel negative regulator of CD73 expression in HCC.

### Co-inhibition of CD73 and A2AR shows therapeutic potential in vitro and in immunodeficient mice

**Fig. 7:**
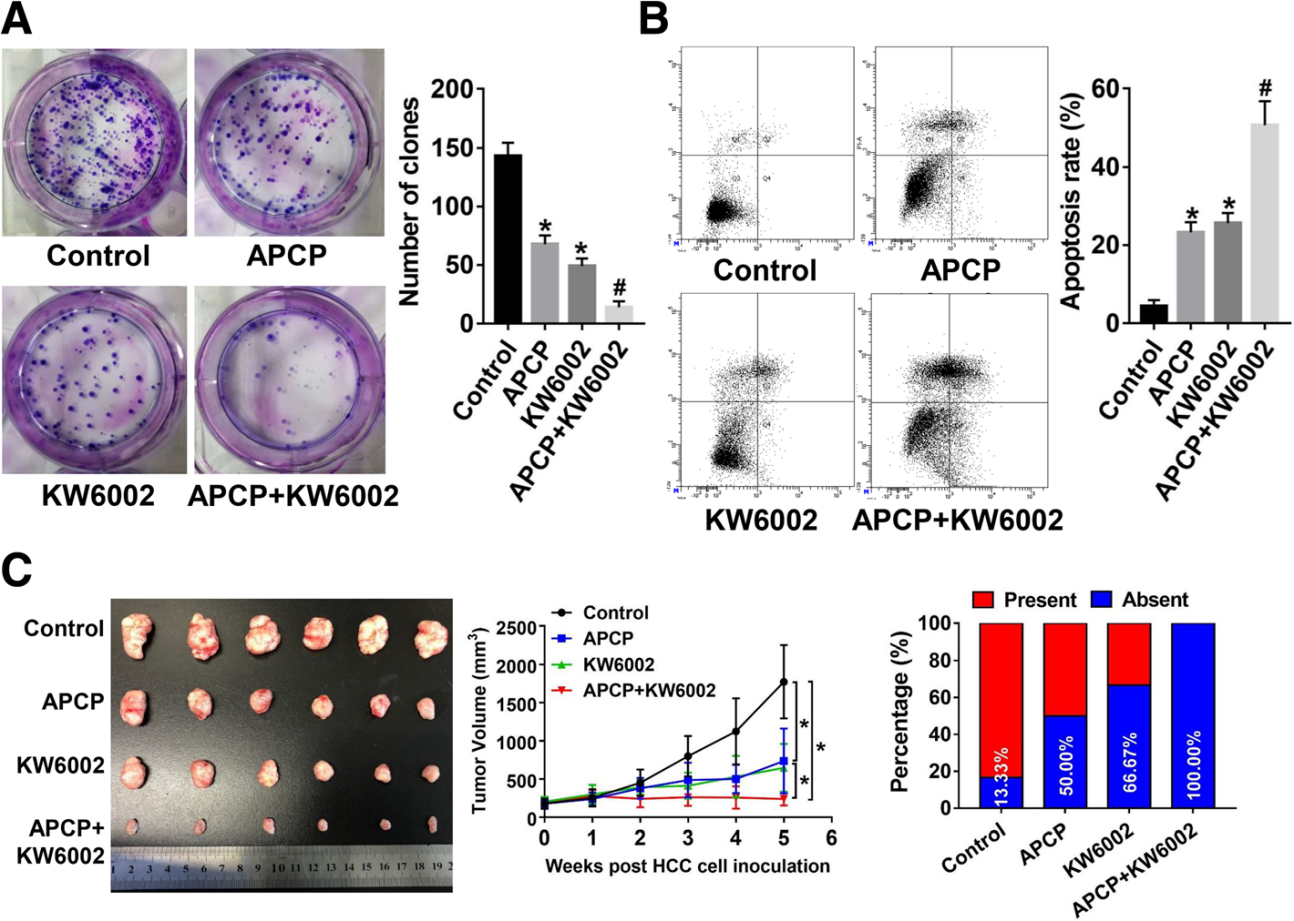
Co-targeting CD73 and A2AR shows therapeutic potential in HCC. **a** Colony formation assays in the indicated HCCLM3 cells. **b** Apoptosis assays in the indicated HCCLM3 cells. **c** Tumor growth kinetics in NOG model mice subcutaneously implanted with HCCLM3 cells treated as indicated. **d** Incidences of lung metastasis of different administered groups versus controls in NOG model mice intravenously injected with HCCLM3 cells. Asterisk indicated *P* < 0.050 when compared with controls; number sign indicated *P* < 0.050 when compared with APCP/KW5002 group alone; all in vitro experiments were performed in triplicate; and *t* tests were used

We examined whether targeting CD73 in combination with A2AR inhibition would inhibit HCC growth independent of the immune system. Colony formation assays showed that co-treatment with inhibitors against CD73 and A2AR dramatically reduced cell proliferation in CD73^high^ HCCLM3 cells, while inhibiting CD73 or A2AR alone showed moderate inhibition effects (Fig. 7a). Moreover, co-targeting strategy induced more apoptosis in HCCLM3 cells than targeting CD73 or A2AR alone (Fig. 7b). Targeting CD73 or A2AR alone in NOG mice caused moderate suppression of tumor growth in vivo. However, dramatic and more durable responses were observed in mice co-treated with both CD73 and A2AR inhibitors (Fig. 7c). Similarly, Targeting CD73 or A2AR alone caused moderate suppression of lung metastasis, while co-targeting strategy exerted a synergistic effect, resulting in maximal suppression of lung metastasis. (Fig. 7d). Our findings revealed co-targeting CD73 and A2AR exhibited potentials for preventing HCC progression and metastasis.

## Discussion

CD73 plays a vital role in adenosinergic signaling by catalyzing AMP into adenosine [10]. Hence, CD73 has been mainly studied for its immunosuppression functions and was identified as a novel immune checkpoint target with promising potential for suppressing tumor development [19, 35]. Meanwhile, studies also showed CD73 promotes tumor growth in an immune-independent manner [16, 17, 30, 31]. Here, functional experiments demonstrated that CD73 could promote HCC progression and metastasis. We also confirmed the clinical significance of CD73 as an independent prognostic indicator of TTR and OS for HCC patients after curative resection. Moreover, CD73 retained significant prognostic prediction value in early-HCC and AFP-low patients, whose clinical outcomes were difficult to predict by conventional indexes [24]. Our findings collaborated with the prognostic significance of CD73 in TCGA database (Additional file 12: Figure S11) and indicated that it might serve as an indicator to identify HCC patients at high risk of recurrence that require further interventions in addition to resection to improve their prognosis.

EMT plays an important role in facilitating HCC metastasis, despite its controversial function during carcinogenesis [36]. Previous studies reported CD73 as a critical regulator for the maintenance of mesenchymal traits in mesenchymal stem cells and ovarian cancer cells [16, 37, 38]. In the present study, we showed that CD73 expression was essential for maintaining the mesenchymal-like phenotype in HCC. Clinical samples confirmed our findings, showing that CD73 expression significantly correlated with EMT status. Therefore, we provide the first evidence demonstrating that CD73 serves as a trigger for EMT in HCC.

Previous studies reported that CD73 exerted its functions by both enzymatic-dependent and enzymatic-independent ways [15]. Here, we found that CD73 exerts its tumor promotion functions mainly through its enzymatic activity due to the significant changes in HCC cells after APCP or adenosine treatment. However, it should be noted that APCP showed a weaker effect on cell proliferation and invasion than CD73, although this difference did not reach a statistical significance. Similar results were observed when comparing adenosine with CD73 overexpression. These findings suggested the possibility of other regulatory mechanisms of CD73 beyond producing adenosine. Thus, our future work will examine the non-enzymatic functions of CD73 to elucidate the regulatory network of CD73 in HCC.

A2AR is a critical receptor that is responsible for the function of CD73 in cancer immunity, and CD73-expressing cancer cells are prone to form metastasis loci via activation of A2AR, which results in exhaustion of natural killer cells within the tumor microenvironment [8, 13, 14, 19]. Here, we provided the first evidence that A2AR serves as the key downstream mediator of CD73 to regulate the progressive phenotype of HCC, and inhibition of A2AR dramatically abolished the effects of CD73 on HCC cells. Our data strongly demonstrate the non-immunosuppressive effects of A2AR, thus expanding our understanding of the function of A2AR in HCC development. Based on these findings, we speculated that co-targeting CD73 and A2AR might achieve satisfactory treatment effects in HCC. Indeed, CD73 inhibition in combination with A2AR blockade resulted in significantly decreased tumor growth as well as metastasis compared with controls. Since our findings were observed in immunodeficient mouse models, we hypothesize that this co-inhibition strategy might achieve more promising treatment response by not only blocking progressive traits of HCC but also restoring anti-tumor immune function within the tumor microenvironment.

Abnormal AKT activation is a hallmark of tumor progression in various cancers, including HCC [39]. In the present study, Alterations of pGSK3β and pAKT levels were observed. More importantly, FOXO3a, a well-established tumor suppressor in HCC according to previous study [40], was also greatly repressed due to CD73 expression, which served as solid evidence for AKT activation. Our findings were consistent with previous studies that CD73 activated AKT in breast and lung cancer cells [17, 30]. However, the underlying mechanism of how CD73 triggered AKT activation had not been elucidated clearly by previous studies. Here, we showed that the CD73-A2AR axis served as a “switch” for Rap1 activation, thus resulting in membrane localization of P110β and PIP3 production, which are critical steps for AKT activation. Rap1 is a central regulator of cell adhesion and motility, and aberrant activation of Rap1 can result in carcinogenesis and tumor progression [27]. Importantly, Rap1 and Ras share similar binding partners, including PI3K [41]. Our study not only confirmed the importance of Rap1 in HCC progression and metastasis, but also identified Rap1 as a key downstream responder of CD73-A2AR that recruits P110β to the plasma membrane. It was reported that cAMP acted as a key downstream molecule of GPCR and could induce Rap1 activation [42]. Based on our Cignal pathway screening results, we also found that cAMP/PKA signaling exhibited significant changes due to CD73 alteration (Fig. 4a). Hence, A2AR might mediate Rap1 activation through promoting cAMP production, and detailed mechanism is currently ongoing in our lab. Previous reports also showed that CD73 was positively correlated with EGFR [22], and future studies should also examine their potential interaction in HCC.

Regulatory network of miRNA-mRNA interaction was important for HCC progression. miR-193a was reported as a tumor suppressor and could be found in HCC cell-derived exosomes [33]. However, miR-193b did not serve as a significant prognostic indicator from HCC-derived exosomes. The major explanation for this might be the different expression regulatory mechanism of these two miRNAs in HCC: low miR-193b expression might due to inhibition of transcription while low miR-193a level might result from selective packaging and secreting through exosomes. Therefore, it was rational that miR-193b could rarely be found in HCC-derived exosomes. Meanwhile, CD73 expression showed little response to miR-193a mimics transfection in our study. We speculated this phenomenon might result from different clearance mechanism of exogenous miRNA between miR-193a and miR-193b. However, our findings demonstrated that miR-193b was the major negative regulator of CD73 in HCC, and our future work will aim at clarifying the underlying mechanism of inhibitory difference between miR-193a and miR-193b.

## Conclusions

Our study demonstrated that CD73 was an independent prognostic indicator for HCC. Functional experiments established the essential role of CD73 in promoting HCC progression and metastasis through PI3K/AKT signaling by inducing Rap1-mediated membrane localization of P110β. We also identified miR-193b as a potential upstream regulator of CD73 in HCC. Notably, co-targeting CD73 and A2AR achieved satisfactory inhibitory effects on HCC in vitro and vivo. Together these results demonstrate the importance of CD73 in HCC in addition to its immunosuppressive functions and revealed that a CD73 targeting strategy may be a promising novel therapeutic strategy for future HCC management.

## Additional files

Additional file 1: Supplementary methods and materials. (DOCX 28 kb)

Additional file 2: **Figure S1.** Prognostic value of CD73 in subgroups of HCC patients. (A) Kaplan–Meier analysis of TTR of early-stage (BCLC 0+A) patients after curative resection according to CD73 expression level. (B) Kaplan–Meier analysis of OS of early-stage (BCLC 0+A) patients after curative resection according to CD73 expression level. (C) Kaplan–Meier analysis of TTR of curative resection according to CD73 expression level. (D) Kaplan–Meier analysis of OS of low-AFP patients after curative resection according to CD73 expression level. (TIF 1853 kb)

Additional file 3: **Figure S2.** Function of CD73 in Hep3B cell line. (A) Efficiencies of CD73 knockdown in Hep3B cells were evaluated by WB assays. (B) Effects of CD73 knockdown on proliferation in Hep3B cells were evaluated by CCK-8 and colony formation assays. (C) Effects of CD73 knockdown on cell cycle in Hep3B cells were evaluated by flow cytometry assays. (D) Effects of CD73 knockdown on apoptosis in Hep3B cells were evaluated by flow cytometry assays. (E) Effects of CD73 knockdown on migration and invasion in Hep3B cells were evaluated by Transwell assays. (F) Effects of CD73 knockdown on migration were validated by wound healing assays. (G) Effects of CD73 knockdown on in vivo tumor growth. “N.S.” indicated not significant; Asterisk indicated *P* < 0.050, all in vitro experiments were performed in triplicate, *t* tests were used. (TIF 10457 kb)

Additional file 4: **Figure S3.** Effects of CD73 on cell motility. (A) Representative images of Transwell assays conducted with control or CD73 knockdown (KD) HCCLM3 cells. (B) Representative images of Transwell assays conducted with control or CD73 overexpression (OE) SMMC7721 cells. (C) Representative images of wound healing assays conducted with control or CD73-KD HCCLM3 cells. (D) Representative images of wound healing assays conducted with control or CD73-OE SMMC7721 cells. “N.S.” indicated not significant; Asterisk indicated *P* < 0.050, all in vitro experiments were performed in triplicate, *t* tests were used. (TIF 13202 kb)

Additional file 5: **Figure S4.** Biological effects of CD73 overexpression in HCCLM3 cells. (A) Efficiencies of overexpression of CD73 in HCCLM3 cells were validated by RT-PCR (left) and WB assays (right). (B) Effects of CD73 overexpression on proliferation were evaluated by CCK-8 (left) and colony formation (right) assay. (C) Effects of CD73 overexpression on migration and invasion were evaluated by Transwell assays. (D) Effects of CD73 overexpression on migration were validated by wound healing assays. (E) Effects of CD73 overexpression on cell survival were evaluated by flow cytometry. (F) Effects of CD73 overexpression on cell cycle were evaluated by flow cytometry. (G) Effects of CD73 overexpression on E-cadherin, N-Cadherin, and pAKT level were evaluated by WB assays. Asterisk indicated *P* < 0.050, all in vitro experiments were performed in triplicate, *t* tests were used. (TIF 5605 kb)

Additional file 6: **Figure S5.** In vivo function of CD73 in HCC. (A) Representative IHC images of PCNA expression level in indicated HCCLM3 (left) or SMMC7721 cells (right). (B) Representative image of intrahepatic metastasis. (C) Representative images of lung metastasis in mice implanted with HCCLM3 or SMMC7721 cells. (TIF 10892 kb)

Additional file 7: **Figure S6.** Role of PI3K/AKT signaling in CD73 mediating HCC progression. (A) Expressions of EMT-related markers in the indicated HCC cells were detected by RT-PCR assays; asterisk indicated *P* < 0.050, experiments were performed in triplicate, and *t* tests were used. (B) Representative images of colony formation assays of the indicated HCCLM3 (upper) and SMMC7721 cells (lower). (C) Representative results of apoptosis assays in the indicated HCCLM3 (upper) and SMMC7721 cells (lower). (D) Representative images of Transwell assays of indicated HCCLM3 (upper) and SMMC7721 cells (lower). (TIF 9692 kb)

Additional file 8: **Figure S7.** CD73 function in HCC depends on its enzymatic activity. (A) Proliferation capacity in the indicated HCCLM3 detected by CCK-8 assays. (B) Invasion capacity in the indicated HCCLM3 detected by Transwell assays. (C) Expressions of EMT-related markers in indicated HCCLM3 cells were detected by RT-PCR. (D) Proliferation capacity in the indicated SMMC7721 detected by CCK-8 assays. (E) Invasion capacity in the indicated SMMC7721 detected by Transwell assays. (F) Expressions of EMT-related markers of indicated SMMC7721 cells were detected by RT-PCR. “N.S.” indicated not significant; asterisk indicated *P* < 0.050, all in vitro experiments were performed in triplicate, and *t* tests were used. (TIF 4551 kb)

Additional file 9: **Figure S8.** CD73 function in HCC occurs through adenosine receptor A2A. (A) Proliferation capacity in HCCLM3 cells treated with indicated adenosine receptor antagonists detected by CCK-8 assays. (B) Invasion capacity in HCCLM3 cells treated with indicated adenosine receptor antagonists detected by Transwell assays. (C) Expressions of EMT-related markers in HCCLM3 cells treated with indicated adenosine receptor antagonists were detected by RT-PCR. (D) Proliferation capacity in Hep3B cells treated with indicated adenosine receptor antagonists detected by CCK-8 assays. (E) Invasion capacity in Hep3B cells treated with indicated adenosine receptor antagonists detected by Transwell assays. (F) Expressions of EMT-related markers of Hep3B cells treated with indicated adenosine receptor antagonists were detected by RT-PCR. Asterisk indicated *P* < 0.050, all in vitro experiments were performed in triplicate, and *t* tests were used. (TIF 5733 kb)

Additional file 10: **Figure S9.** Role of Rap1 activation in AKT phosphorylation. (A) Expressions of P110α, P110β, pAKT, and AKT in HCCLM3 cells were detected by WB assays. (B) Expressions of P110α, P110β, pAKT, and AKT in Hep3B cells were detected by WB assays. (C) Expressions of pAKT and AKT in 293T transfected with indicated plasmids were detected by WB assays. (D) Cellular PIP2 and PIP3 levels of 293T transfected with indicated plasmids were detected by ELISA assays. “N.S.” indicated not significant; asterisk indicated *P* < 0.050, experiments were performed in triplicate, and *t* tests were used. (TIF 725 kb)

Additional file 11: **Figure S10.** miR-193b served as an upstream negative regulator for CD73 expression in HCC. (A) Prediction results of microRNAs that potentially regulate CD73 according to StarBase 2.0. (B) Effects of miR-193a mimics transfection on CD73 expression in HCC cells were evaluated by RT-PCR and WB assays. (C) Representative images of Transwell assays in indicated HCCLM3 (upper) and Hep3B (lower) cells. (D) Migration evaluation via wound healing assays of miR-193b mimic-transfected HCC cells with or without CD73 overexpression. All in vitro experiments were performed in triplicate, and *t* tests were used. (TIF 6462 kb)

Additional file 12: Figure S11 Clinical significance of CD73 in HCC according to TCGA database. Data was collected from The Human Protein Atlas (https://www.proteinatlas.org/ENSG00000135318-NT5E/pathology/tissue/liver+cancer). (TIF 378 kb)

## Abbreviations

A2AR: Adenosine A2A receptor
BCLC: Barcelona Clinic Liver Cancer
co-IP: Co-immunoprecipitation
CTC: Circulating tumor cell
DMEM: Dulbecco’s modified Eagle’s medium
EMT: Epithelial-mesenchymal transition
HCC: Hepatocellular carcinoma
HR: Hazard ratio
NOG: NOD/SCID/γc(null)
OS: Overall survival
shRNA: Short hairpin RNA
TTR: Time to recurrence
WB: Western blot
