# Supplementary material for: CD73 promotes hepatocellular carcinoma progression and metastasis via activating PI3K/AKT signaling by inducing Rap1-mediated membrane localization of P110β and predicts poor prognosis
Source: J Hematol Oncol. 2019 Apr 11;12:37. doi: 10.1186/s13045-019-0724-7 (PMC6458749; doi:10.1186/s13045-019-0724-7)
Supplement: Supplementary file 1 — Supplementary methods and materials. (DOCX 28 kb) [file 13045_2019_724_MOESM1_ESM.docx]

**Supplementary Methods and Materials**

**HCC cell lines**

MHCC97H, MHCC97L, HCCLM3, Huh7, SMMC7721, and HepG2 cell lines were obtained from the Liver Cancer Institute, Fudan University, Shanghai, China. The Hep3B cell line was purchased from the cell bank at the Institute of Biochemistry and Cell Biology, China Academy of Science (Shanghai, China). Cells were cultured in high-glucose Dulbecco’s modified Eagle’s medium (DMEM) supplemented with 10% fetal bovine serum (FBS) and 1% penicillin and 100 μg/ml streptomycin in a humidified incubator containing 5% CO_2_ at 37ºC. All experiments were performed in cells less than 8 passages. All reagents for cell culture were purchased from Gibco (Thermo, USA).

**Cell transfection**

shCD73 Oligos were cloned into the pLVX-Puro vector (GeneChem, Shanghai). Lentivirus was generated using the packaging cell line HEK293T, purchased from the cell bank at the Institute of Biochemistry and Cell Biology, China Academy of Science (Shanghai, China) and cultured as described previously[1]. The viral supernatant was harvested 48 h post-transfection. After passing through 0.45 μM syringe filters, an appropriate amount of viruses was used to infect target cells in the presence of 8 μg/ml polybrene. The sequences of shCD73-1 was 5′-CCTAGGCTATCTGAAGATC-3′, and shCD73-2 was 5′-AGCAGCATTCCTGAAGATC-3’. For CD73 overexpression, an expression plasmid pLVX-CD73 was designed and synthesized by Genechem lab (Shanghai, China). Cells infected with viruses underwent selection with 1 μg/ml of puromycin.

**Antibodies and reagents**

The following antibodies were used in present study: anti-CD73 (Abcam), anti-E-Cadherin (Cell Signaling Technology, CST), anti-N-Cadherin (CST), anti-Vimentin (CST), anti-Twist (Abcam), anti-phospho-AKT (pAKT, at Ser473, Abcam), anti-AKT (Abcam), anti-phospho-GSK3β (pGSK3β, at Ser 9, Abcam), anti-GSK3β (Abcam), anti-Cyclin D1 (CCND1, CST), anti-Snail (Abcam), anti-Rap1 (Abcam), anti-p110α (Abcam), anti-p110β (Abcam), anti-Na^+^-K^+^ ATPase (Abcam), FOXO3a (Abcam), and anti-β-actin (Abcam). The following reagents were used in the present study: MK-2206 (Selleck), SC-79 (Selleck), adenosine (Sigma), α,β-methylene ADP (APCP, Sigma), DPCPX (Abcam), KW6002 (MedChemExpress, MCE), CVT6883 (BOC science), Reversine (Selleck), and CGS21680 (MCE).

**Immunohistochemistry (IHC) staining and TMA evaluations**

TMAs containing samples from 189 HCC patients were constructed as previously described[2]. Before primary antibodies were used on the arrays, the antibodies were titered against normal control tissues to determine the dilutions that rendered optimal sensitivity and specificity. Staining results were visualized by sequential incubations of TMAs with the components of the Envision-plus detection system (EnVision +/HRP/Mo, Dako) and 3,3′-diaminobenidine. Negative controls were treated in the same way except without adding the primary antibodies. The immunoreactive score system was used to evaluate the staining of each sample as follows: staining extent score was on a scale of 0-4, corresponding to the percentage of immunoreactive tumor cells (0%, 1%-5%, 6%-25%, 26%-75%, and 76%-100%, respectively); while staining intensity was scored as negative (score=0), weak (score=1), moderate (score=2), and strong (score=3); immunoreactive score ranking from 0-12 was calculated by multiplying the staining extent score with the intensity score, resulting in a low (0-6) level or a high (8-12) level value for each specimen. IHC staining was assessed by two independent pathologists with no prior knowledge of patient characteristics. Discrepancies were resolved by consensus.

**Follow up**

Post-treatment monitoring was performed as described previously[2]. Briefly, patients were followed up every month during the first 6 months post-treatment and every 3 to 4 months thereafter. All patients were monitored by abdominal ultrasonography and chest X-ray every 1 to 6 months and by computed tomography scans every 6 months. Bone scans or magnetic resonance imaging scans were performed if localized bone pain was reported.

**RT-PCR**

Total RNA was extracted using an RNeasy mini kit (Qiagen, Germany) and cDNA was synthesized using the Quantitect Reverse Transcription Kit (Qiagen) according to the manufacturer’s instructions. Target genes were quantified using FastStart Universal SYBR Green Master (Roche Diagnostics, Germany) and DNA amplification was performed using a LightCycler 480 (Roche Diagnostics, Germany). The relative quantities of target gene mRNAs compared to an internal control were determined using the ΔCq method. PCR conditions were as follows: 5 min at 95°C, followed by 40 cycles of 95°C for 10 s and 60°C for 60 s. GAPDH was used as an internal control. The following primers were used in the present study: CD73, 5′-TTAGGACCTGGCTTTGTG-3′ (F), 5′-GTTGCTGACCCTGAGTAATC-3′ (R); E-Cadherin, 5′-GTAGGAAGGCACAGCCTGTC-3′ (F), 5′-CAGCAAGAGCAGCAGAATCA-3′ (R); Vimentin, 5′-CTGCAGGACTCGGTGGACTT-3′ (F), 5′-GAAGCGGTCATTCAGCTCCT-3′ (R); N-Cadherin, 5′-GAGCATGCCAAGTTCCTGAT-3′ (F), 5′-TGGCCACTGTGCTTACTGAA-3′ (R); Fibronectin, 5′-ACCTGGAGGAGACCACATGA-3′ (F), 5′-CCATCATCCAGCCTTGGTAG-3′ (R); Twist, 5′-GCCGACGACAGCCTGAGCAA-3′ (F), 5′-CGCCACAGCCCGCAGACTTC-3′ (R); Snail, 5′-TCTGAGGCCAAGGATCTCCA-3′ (F), 5′-GTGGCTTCGGATGTGCATCT-3′ (R); CCND1, 5′-GCTGTGCATCTACACCCGACA-3′ (F), 5′-TTGAGCTTGTTCACCAGGAG-3′ (R); and β-actin, 5′-CACCATTGGCAATGAGCGGTTC-3′ (F), 5′-AGGTCTTTGCGGATGTCCACGT-3′ (R).

**Western blot**

Cells were lysed in RIPA lysis buffer (Beyotime, China) containing PMSF (Roche, Germany). The supernatants were collected after centrifugation at 13000 x g at 4°C for 10 min. Protein concentration was determined using a BCA protein kit (Beyotime, China) and whole lysates were mixed with 4× SDS loading buffer (125 mmol/Tris-HCl, 4% SDS, 20% glycerol, 100 mmol/L DTT, and 0.2% bromophenol blue) at a ratio of 1:3. Protein samples (30 μg) were heated at 100°C for 15 min, separated by SDS-polyacrylamide gel electrophoresis, and transferred onto a polyvinylidene fluoride membrane. The membrane was incubated first with primary antibodies at 4°C, followed by another 2 h incubation with secondary antibody (1:5000). β-actin served as a loading control. The secondary antibodies were horseradish peroxidase-conjugated anti-rabbit antibodies (Jackson ImmunoResearch Labs, West Grove, PA, USA). Densitometric analysis of the western blots was performed with NIH Image J software (Bethesda, MD, USA). The protein levels were first normalized to β-actin and subsequently to the experimental controls.

**Circulating tumor cell (CTC) load evaluation**

CTC loads were determined using an optimized CTC detection platform established by our previous study[3]. A cutoff value of 0.8 was set to stratify patients into CTC^high^ (CTC load > 0.8/5 ml) and CTC^low^ (CTC load ≤ 0.8/5 ml) subgroups according to our previous study[3].

**Proliferation assays**

For CCK-8 assays, cells (2000 cells/100 μl per well) were seeded in a 96-well plate. Viable cells were assessed after 24, 48, and 72 h according to the manufacturer’s instructions. Briefly, cells were incubated in 10% CCK-8 (Dojindo, Japan) diluted in normal culture medium for an additional 2 h. The absorbance at a wavelength of 450 nm was used to estimate viable cells in each well. For colony formation assays, HCC cells were seeded at a density of 1000 cells per well in 6-well plates and maintained in complete medium containing 10% FBS (Gibco, USA) for 14 days. Colonies were fixed with methanol and stained with Giemsa staining solution (Sigma, USA). Visible colonies were photographed and counted manually.

**Migration and invasion assays**

Transwell assays were performed to evaluate migration and invasion activities. Cells were collected and washed with 1xPBS. For migration assays, 5 x 10^4^ cells were seeded in the upper chamber with a non-coated membrane (24-well insert, pore size 8 μm; Corning, USA) with DMEM containing 1% FBS. For invasion assays, 5 x 10^4^ cells were seeded in the upper chamber with a MatriGel-coated membrane (dilution: 1:8). In both assays, the lower chambers contained DMEM with 10% FBS as chemo-attractant. Cells were incubated at 37°C for 24 h. Cells that had migrated or invaded to the lower surface of membrane were fixed by 4% methanol, followed by staining with crystal violet. Stained cells were counted in 10 random 100X microscopic fields. All experiments were conducted in triplicate. The unpaired two-tail student’s t-test was used for comparison.

**Wound healing assay**

Wounding healing assay was conducted as previous described[4]. Briefly, cells were washed with PBS for 3 times after, and cultured with DMEM supplemented with 1% FBS. All assays were performed in triplicate.

**Flow cytometry**

For cell cycle analysis, the FxCycle Violet Stain kit (Invitrogen, USA) was used according to the manufacturer’s instructions. Briefly, cells were harvested and fixed using 4% methanol. After three washes, the cell concentration was adjusted to 1 x 10^6^ cells per ml. Then, 1 μl of FxCycle Violet stain was added to each sample, and cells were incubated for 30 min at room temperature, protected from light. Cells were analyzed using an Aria II flow cytometer (BD, USA). For apoptosis analysis, the FITC Annexin V Apoptosis Detection Kit II (BD, USA) was used according to the manufacturer’s instructions. Briefly, cells were harvested and washed with pre-cold PBS buffer three times. Then, 5 μl of FITC-labeled Annexin V and 5 μl of PI solution were added to each sample, and cells were incubated for 15 min at room temperature. Cells were analyzed using the Aria II flow cytometer.

**Immunofluorescence**

For EMT-related markers staining, cells were fixed in 4% paraformaldehyde and blocked with 5% bovine serum albumin. Samples were incubated with phycoerythrin (PE)-conjugated mouse anti-human E-Cadherin (BD, USA), N-Cadherin (BD, USA) or Vimentin (BD, USA) overnight at 4°C. After washing with PBS three times, cells were counterstained with DAPI (Sigma-Aldrich, USA). For Rap1 and P110β staining, cells were incubated with mouse anti-Rap1 (1:100, Abcam) and rabbit anti-P110β (1:100, Abcam) primary antibodies overnight at 4°C. After washing with PBS three times, cells were stained by PE-conjugated goat anti-rabbit (CST, 1:500) and FITC-conjugated goat anti-mouse secondary antibodies (Abcam, 1:500) for 2 hours at room temperature. Afterwards, cells were counterstained with DAPI (Sigma-Aldrich, USA). For cytoskeletal staining, cells were firstly fixed in 4% paraformaldehyde and blocked with 5% bovine serum albumin. Then, cells were stained with 488-conjugated phalloidin (Abcam) for 1 hour at room temperature, and cells were counterstained with DAPI for final evaluations.

**Cytosolic and plasma membrane fractionation**

Isolation of cytosolic and membrane proteins was conducted according to previous studies[4, 5]. Briefly, cells were harvested and washed with pre-cold PBS buffer for three times and then resuspended on ice in a hypotonic buffer. Cells were sheared and lysates were centrifuged at a low speed. After discarding the precipitation (nuclei), the supernatant was re-centrifuged. Both supernatant (cytosolic fractions) and precipitation (membrane fractions) were harvested. Membrane fractions were washed twice with hypotonic buffer and resuspended on ice in lysis buffer containing 1% NP-40. Protein concentrations of both fractions were determined using Bradford assay (Beyotime, China).

**Rap1 knockdown and Rap1-GTP quantification**

Rap1 knockdown was conducted using a shRNA plasmid targeting human Rap1 (Santa Cruz, USA) according to manufacturer’s instructions. After indicated manipulations, cells were lysed on ice according to previous studies. After centrifugation, protein concentrations were determined with the BCA Protein Assay Kit (Beyotime, China). To quantify the active form of Rap1, Rap1-GTP, the Active Rap1 Detection Kit (CST, USA) was used according to the manufacturer’s instructions, and WB was performed with anti-Rap1 antibodies (Abcam, USA).

**In vivo xenograft experiments**

To minimize the influence of the immune system in our study, supra-immunodeficient NOD/SCID/γc(null) (NOG) mice, purchased from the Chinese Academy of Medical Science, were used for experiments. Establishment of orthotopic liver cancer model mice was conducted according to previous studies[5]. In brief, 5 × 10^6^ cells were injected subcutaneously into the left upper flank regions of NOG mice. The subcutaneous tumor tissues were removed 6 weeks later, cut into pieces of the same size (1 mm^3^), and implanted into the liver of each group respectively (6 in each group). At 8 weeks after implantation, the mice were sacrificed. Tumor sizes were calculated using a vernier caliper as follows: tumor volume (mm^3^) = (L × W2)/2, where L = long axis and W = short axis. To evaluate lung metastasis incidence, all lung tissues from xenografts were harvested, fixed with 4% paraformaldehyde, embedded, and received consecutive resection, followed by HE staining. Lung metastases were carefully evaluated by two senior histopathologists.

For inhibitor experiments, the subcutaneous NOG mouse models were established as described. Once the xenograft size reached approximately 5 × 5 mm^3^ (length × width), the mice were randomly divided into four groups: (1) 0.05% DMSO control (administrated i.p. every other day); (2) APCP (15 mg/Kg, i.p. every other day); (3) KW6002 (10 mg/Kg, p.o. every other day); and (4) 15 mg/Kg APCP (i.p.) plus 10 mg/Kg KW6002 (p.o.) (n=6/group). Tumor growth was recorded for 5 weeks.

To evaluate the effects of inhibitor treatment on preventing metastasis, NOG mice were injected intravenously (via tail veins) with 1 × 10^5^ HCCLM3 cells to establish lung metastasis models to exclude the influence of tumor growth in orthotopic models (Day 0). The mice were randomly divided into four groups and treated as described above. After 20 days, mice were sacrificed and lung tissues were harvested for metastasis evaluation as described.

**Co-****immunoprecipitation (co-IP)**

Co-IP assay was performed according to previous studies[6, 7]. Briefly, HEK-293T cells were transfected with expression plasmids. At 48 later, lysates were prepared using lysis buffer containing 20 mM Tris-HCl (pH 8.0), 150 mM NaCl, 0.5% NP-40, 2.5mM EDTA, and protease inhibitor mixture. Lysates were pre-cleared with protein A/G beads, and co-IP assays were performed with either HA (CST, USA) or Flag (CST, USA) antibodies. Endogenous Rap1 and P110β binding was evaluated according to a previous study. In brief, lysates were incubated with anti-Rap1 antibody (Abcam, USA) and protein A/G agarose gel overnight. After washing with RIPA buffer for 4–6 times, the proteins coupled to the gel were boiled in protein loading buffer, followed by WB analysis.

**Cellular phosphoinositide measurement**

Lipid extraction and cellular phosphoinositide measurement were conducted using PI(3,4,5)P3 Mass ELISA and PI(4,5)P2 Mass ELISA Kit (Echelon Biosciences) according to manufacturer’s instructions. Recovered lipids were dried, followed by competitive ELISA analysis. PI(4,5)P_2_ levels were used as a control for total phosphoinositides as it is the most abundant phosphoinositide and it is not affected by PI3K activity.

**Luciferase reporter assay**

Interactions between miR-193b and CD73 in HCC were verified by luciferase assay according to previous studies. Briefly, HCCLM3 and Hep3b cells plated in 24-well plates were co-transfected with psiCHECK-2-CD73–3’-UTR reporter and 5 pmol of miR-193b mimics. pRL-TK (Promega, USA) was used as a normalization control. After 48 h, cells were harvested and luciferase activity was measured using a dual-luciferase reporter system (Promega, USA).

**References**:

1. Lupia M, Angiolini F, Bertalot G, Freddi S, Sachsenmeier KF, Chisci E, Kutryb-Zajac B, Confalonieri S, Smolenski RT, Giovannoni R, et al: CD73 Regulates Stemness and Epithelial-Mesenchymal Transition in Ovarian Cancer-Initiating Cells. Stem Cell Rep. 2018;10:1412-1425.

2. Hu B, Ding GY, Fu PY, Zhu XD, Ji Y, Shi GM, Shen YH, Cai JB, Yang Z, Zhou J, et al: NOD-like receptor X1 functions as a tumor suppressor by inhibiting epithelial-mesenchymal transition and inducing aging in hepatocellular carcinoma cells. J Hematol Oncol. 2018;11:28.

3. Guo W, Sun YF, Shen MN, Ma XL, Wu J, Zhang CY, Zhou Y, Xu Y, Hu B, Zhang M, et al: Circulating Tumor Cells with Stem-Like Phenotypes for Diagnosis, Prognosis, and Therapeutic Response Evaluation in Hepatocellular Carcinoma. Clin Cancer Res. 2018;24:2203-2213.

4. Yang XR, Xu Y, Yu B, Zhou J, Li JC, Qiu SJ, et al. CD24 is a novel predictor for poor prognosis of hepatocellular carcinoma after surgery. Clin Cancer Res. 2009;15:5518-5527.

5. Xiao S, Chang RM, Yang MY, Lei X, Liu X, Gao WB, Xiao JL, Yang LY: Actin-like 6A predicts poor prognosis of hepatocellular carcinoma and promotes metastasis and epithelial-mesenchymal transition. Hepatology. 2016;63:1256-1271.

6. Xiong Y, Ye C, Yang N, Li M, Liu H: Ubc9 Binds to ADAP and Is Required for Rap1 Membrane Recruitment, Rac1 Activation, and Integrin-Mediated T Cell Adhesion. J Immunol. 2017;199:4142-4154.

7. Meller N, Liu YC, Collins TL, Bonnefoy-Berard N, Baier G, Isakov N, Altman A: Direct interaction between protein kinase C theta (PKC theta) and 14-3-3 tau in T cells: 14-3-3 overexpression results in inhibition of PKC theta translocation and function. Mol Cell Biol. 1996;16:5782-5791.
