# Supplementary figures and images for: CD73 promotes hepatocellular carcinoma progression and metastasis via activating PI3K/AKT signaling by inducing Rap1-mediated membrane localization of P110β and predicts poor prognosis
Source: J Hematol Oncol. 2019 Apr 11;12:37. doi: 10.1186/s13045-019-0724-7 (PMC6458749; doi:10.1186/s13045-019-0724-7)

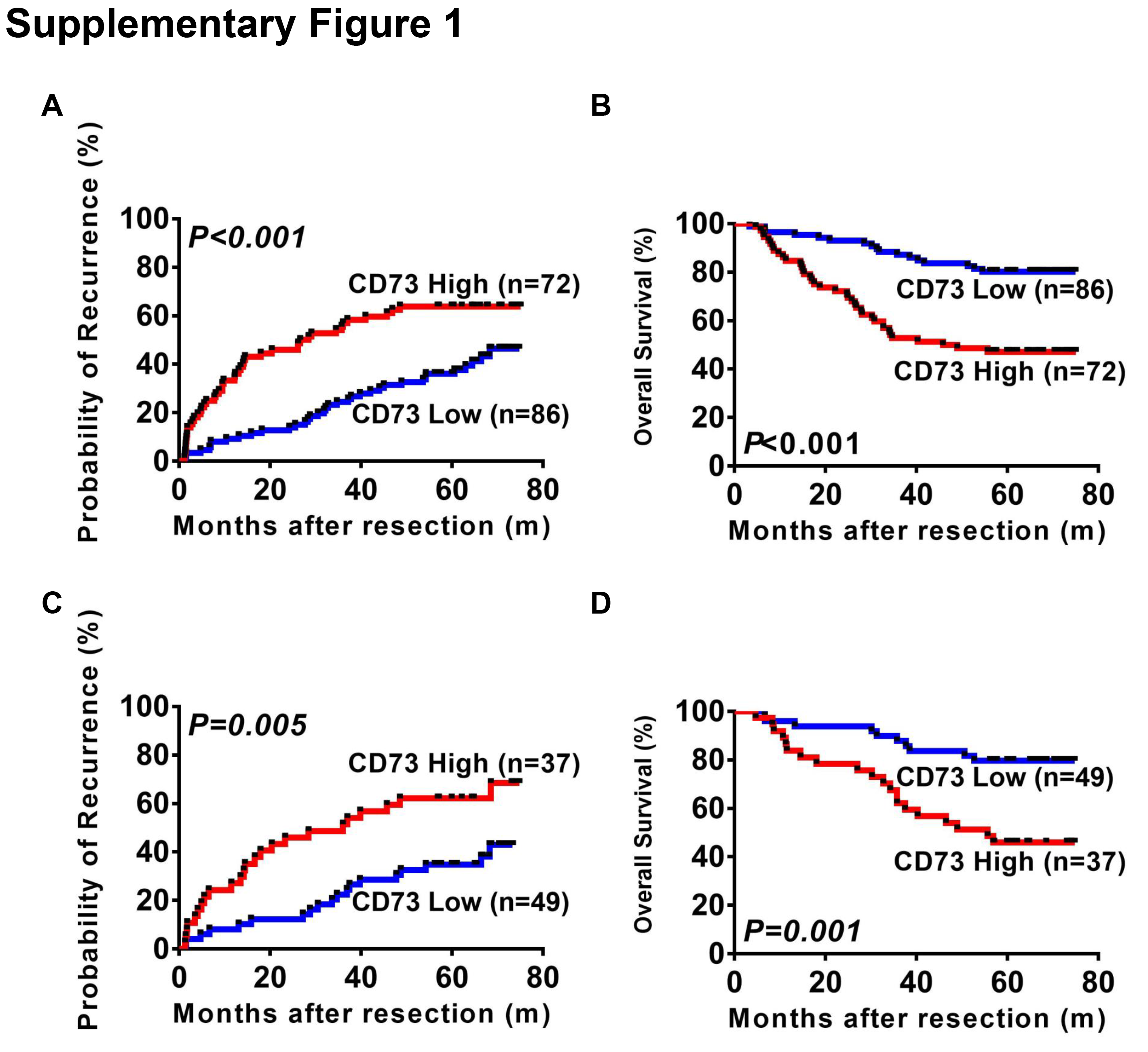

Supplement: Supplementary file 2 — Figure S1. Prognostic value of CD73 in subgroups of HCC patients. (A) Kaplan–Meier analysis of TTR of early-stage (BCLC 0+A) patients after curative resection according to CD73 expression level. (B) Kaplan–Meier analysis of OS of early-stage (BCLC 0+A) patients after curative resection according to CD73 expression level. (C) Kaplan–Meier analysis of TTR of curative resection according to CD73 expression level. (D) Kaplan–Meier analysis of OS of low-AFP patients after curative resection according to CD73 expression level. (TIF 1853 kb) [file 13045_2019_724_MOESM2_ESM.tif]

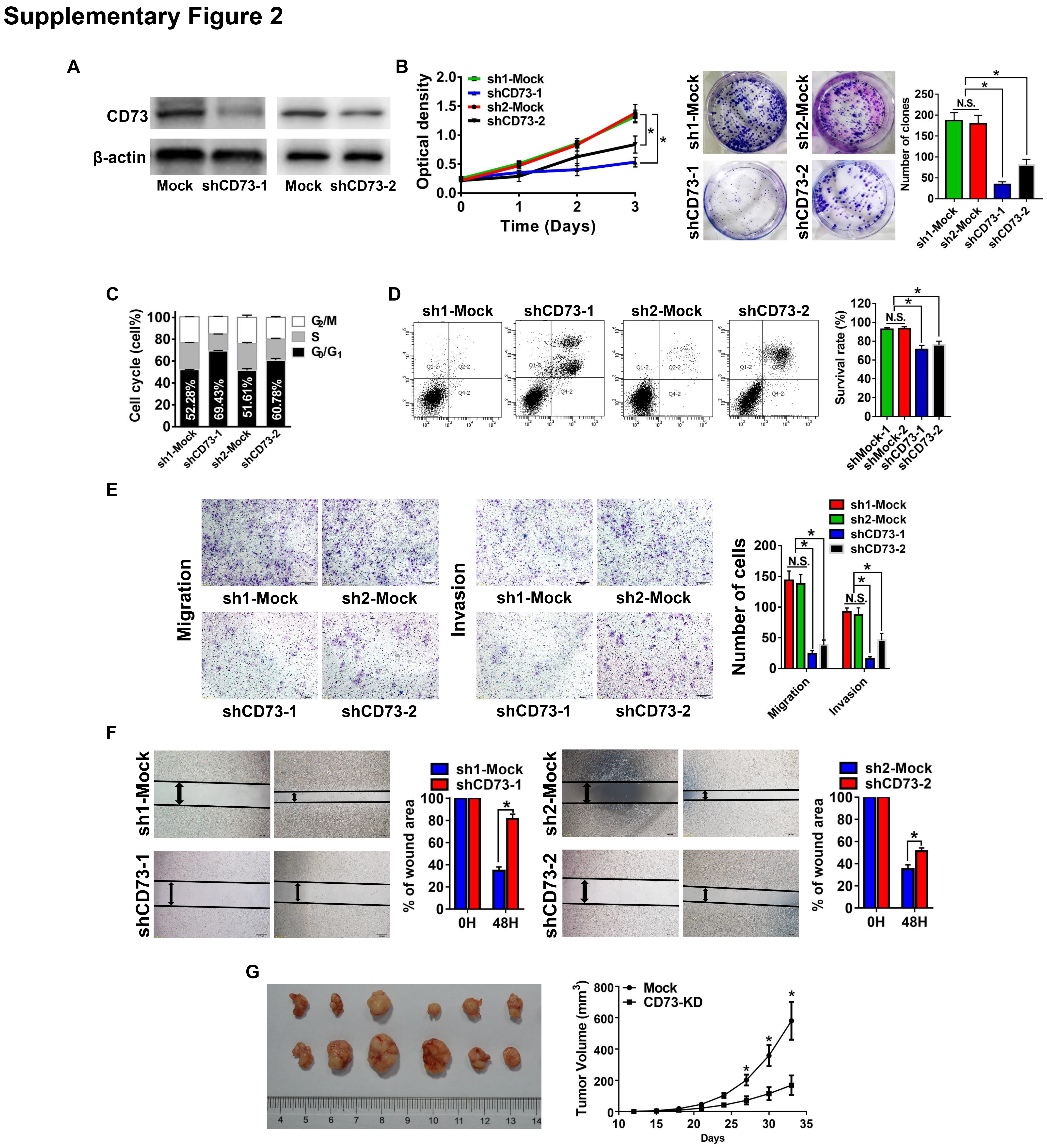

Supplement: Supplementary file 3 — Figure S2. Function of CD73 in Hep3B cell line. (A) Efficiencies of CD73 knockdown in Hep3B cells were evaluated by WB assays. (B) Effects of CD73 knockdown on proliferation in Hep3B cells were evaluated by CCK-8 and colony formation assays. (C) Effects of CD73 knockdown on cell cycle in Hep3B cells were evaluated by flow cytometry assays. (D) Effects of CD73 knockdown on apoptosis in Hep3B cells were evaluated by flow cytometry assays. (E) Effects of CD73 knockdown on migration and invasion in Hep3B cells were evaluated by Transwell assays. (F) Effects of CD73 knockdown on migration were validated by wound healing assays. (G) Effects of CD73 knockdown on in vivo tumor growth. “N.S.” indicated not significant; Asterisk indicated P < 0.050, all in vitro experiments were performed in triplicate, t tests were used. (TIF 10457 kb) [file 13045_2019_724_MOESM3_ESM.tif]

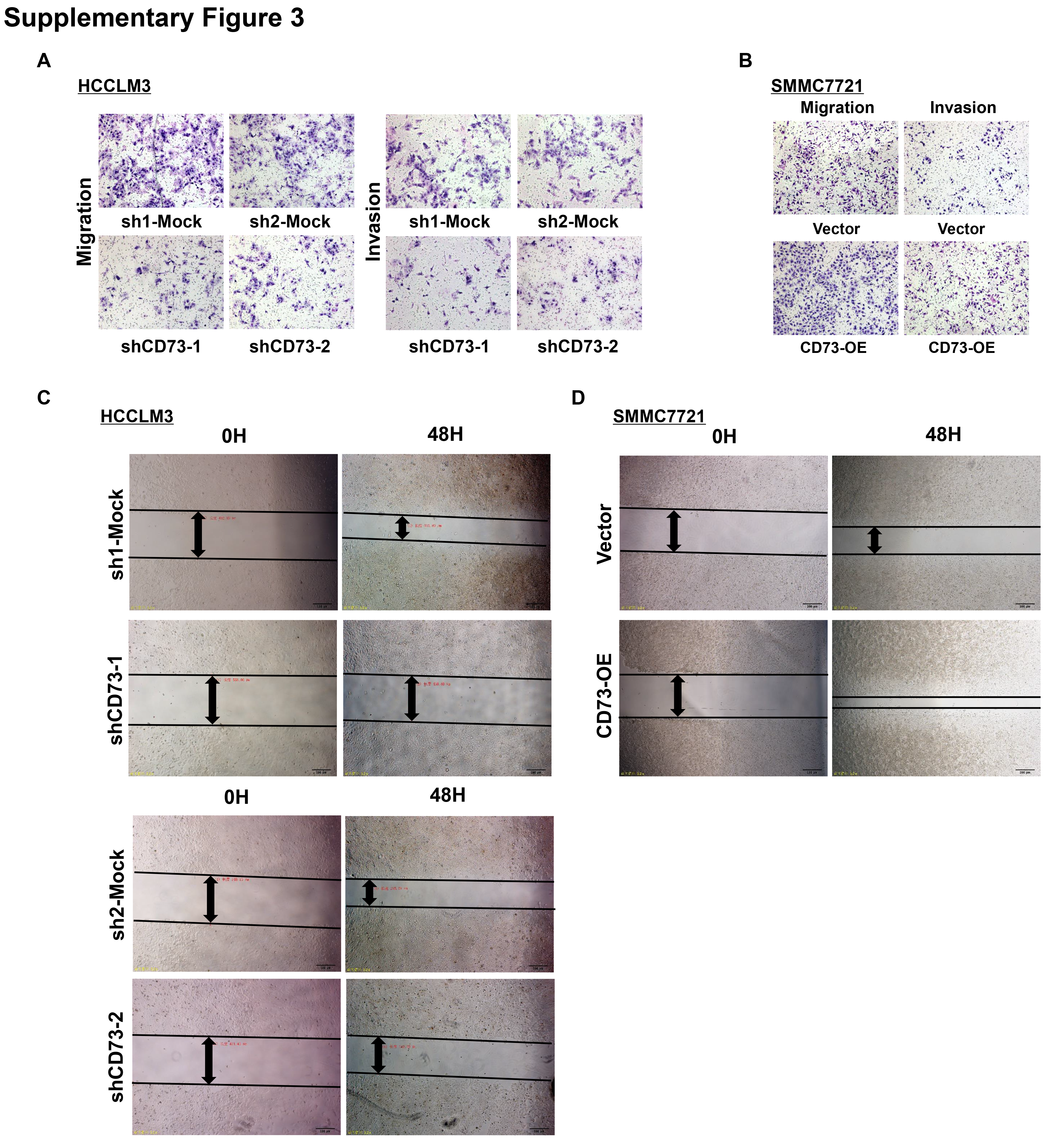

Supplement: Supplementary file 4 — Figure S3. Effects of CD73 on cell motility. (A) Representative images of Transwell assays conducted with control or CD73 knockdown (KD) HCCLM3 cells. (B) Representative images of Transwell assays conducted with control or CD73 overexpression (OE) SMMC7721 cells. (C) Representative images of wound healing assays conducted with control or CD73-KD HCCLM3 cells. (D) Representative images of wound healing assays conducted with control or CD73-OE SMMC7721 cells. “N.S.” indicated not significant; Asterisk indicated P < 0.050, all in vitro experiments were performed in triplicate, t tests were used. (TIF 13202 kb) [file 13045_2019_724_MOESM4_ESM.tif]

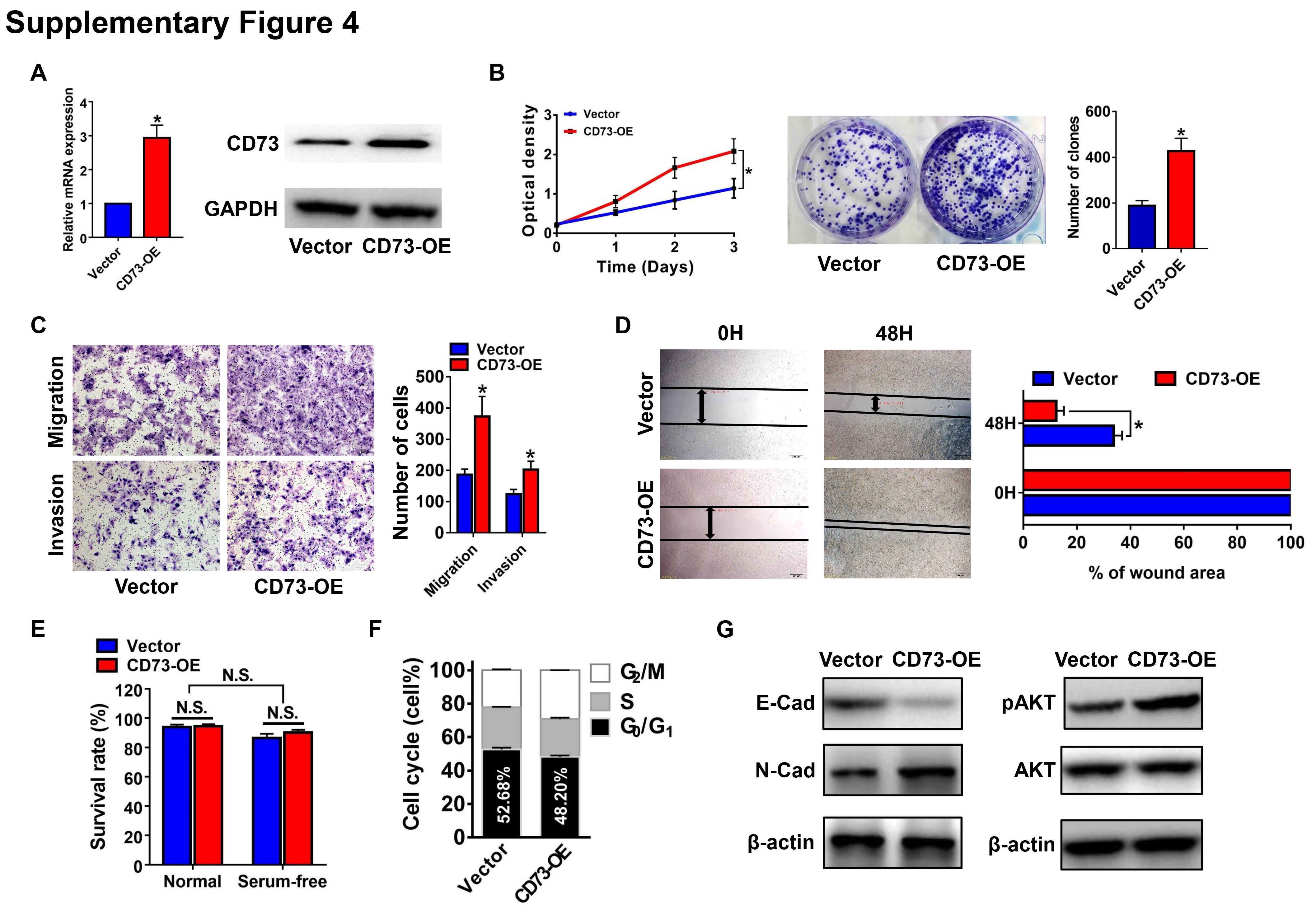

Supplement: Supplementary file 5 — Figure S4. Biological effects of CD73 overexpression in HCCLM3 cells. (A) Efficiencies of overexpression of CD73 in HCCLM3 cells were validated by RT-PCR (left) and WB assays (right). (B) Effects of CD73 overexpression on proliferation were evaluated by CCK-8 (left) and colony formation (right) assay. (C) Effects of CD73 overexpression on migration and invasion were evaluated by Transwell assays. (D) Effects of CD73 overexpression on migration were validated by wound healing assays. (E) Effects of CD73 overexpression on cell survival were evaluated by flow cytometry. (F) Effects of CD73 overexpression on cell cycle were evaluated by flow cytometry. (G) Effects of CD73 overexpression on E-cadherin, N-Cadherin, and pAKT level were evaluated by WB assays. Asterisk indicated P < 0.050, all in vitro experiments were performed in triplicate, t tests were used. (TIF 5605 kb) [file 13045_2019_724_MOESM5_ESM.tif]

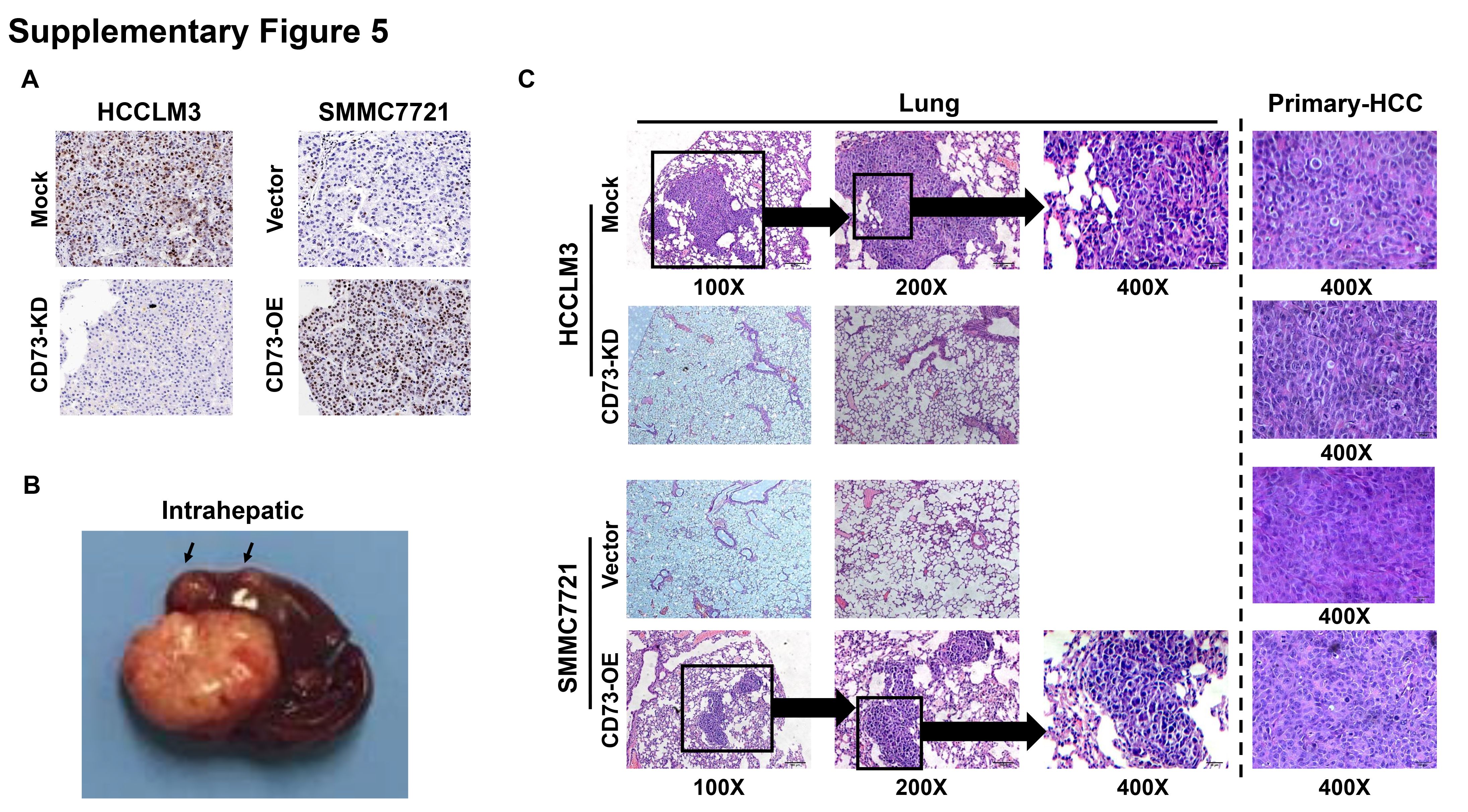

Supplement: Supplementary file 6 — Figure S5. In vivo function of CD73 in HCC. (A) Representative IHC images of PCNA expression level in indicated HCCLM3 (left) or SMMC7721 cells (right). (B) Representative image of intrahepatic metastasis. (C) Representative images of lung metastasis in mice implanted with HCCLM3 or SMMC7721 cells. (TIF 10892 kb) [file 13045_2019_724_MOESM6_ESM.tif]

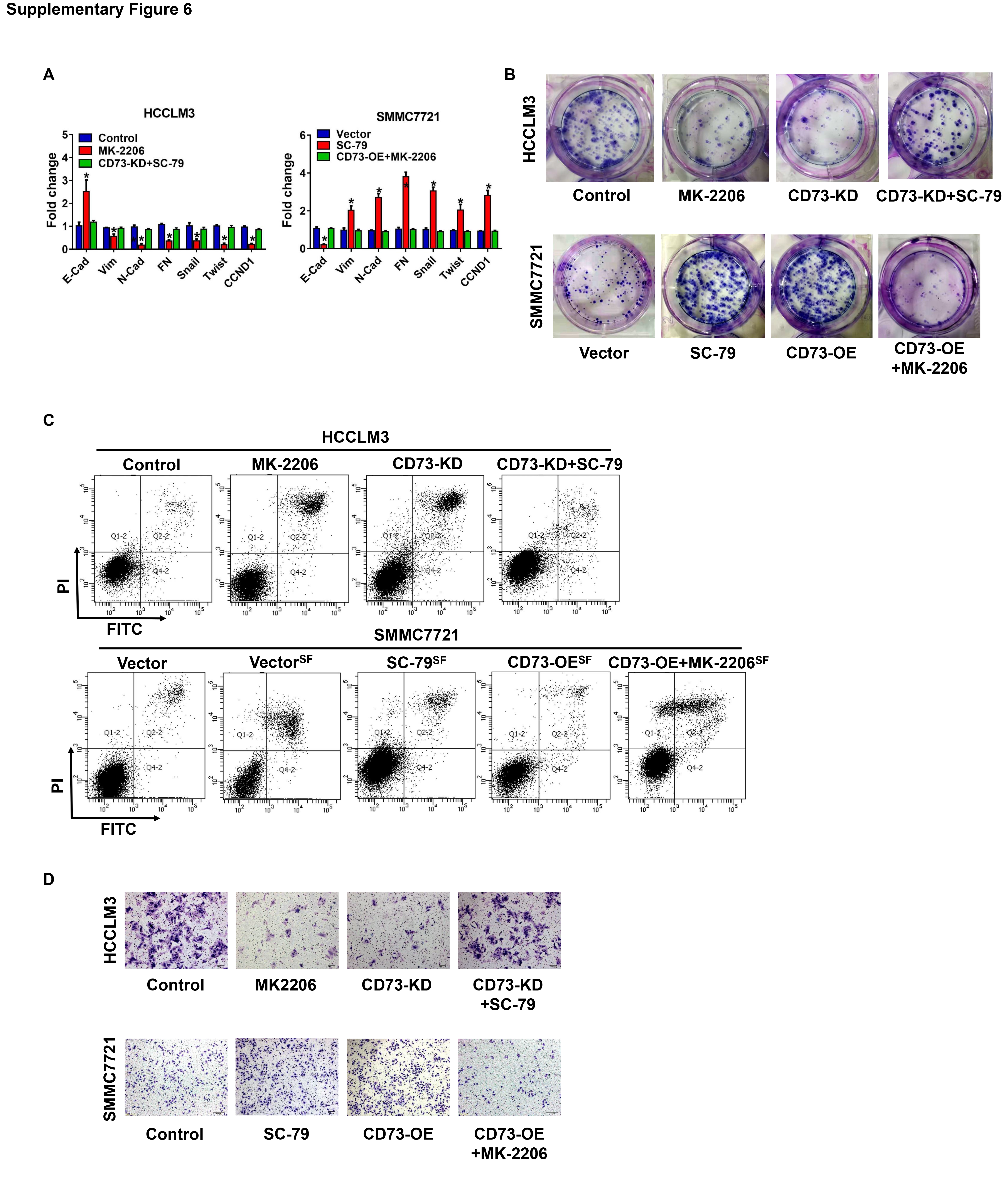

Supplement: Supplementary file 7 — Figure S6. Role of PI3K/AKT signaling in CD73 mediating HCC progression. (A) Expressions of EMT-related markers in the indicated HCC cells were detected by RT-PCR assays; asterisk indicated P < 0.050, experiments were performed in triplicate, and t tests were used. (B) Representative images of colony formation assays of the indicated HCCLM3 (upper) and SMMC7721 cells (lower). (C) Representative results of apoptosis assays in the indicated HCCLM3 (upper) and SMMC7721 cells (lower). (D) Representative images of Transwell assays of indicated HCCLM3 (upper) and SMMC7721 cells (lower). (TIF 9692 kb) [file 13045_2019_724_MOESM7_ESM.tif]

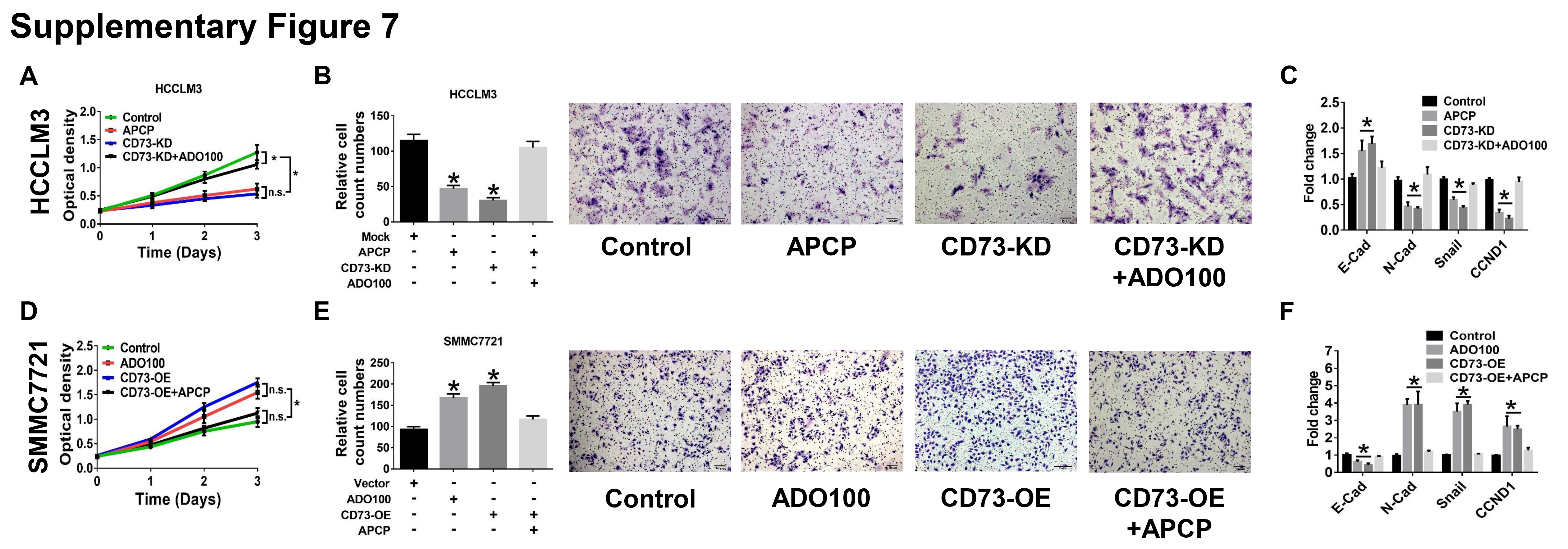

Supplement: Supplementary file 8 — Figure S7. CD73 function in HCC depends on its enzymatic activity. (A) Proliferation capacity in the indicated HCCLM3 detected by CCK-8 assays. (B) Invasion capacity in the indicated HCCLM3 detected by Transwell assays. (C) Expressions of EMT-related markers in indicated HCCLM3 cells were detected by RT-PCR. (D) Proliferation capacity in the indicated SMMC7721 detected by CCK-8 assays. (E) Invasion capacity in the indicated SMMC7721 detected by Transwell assays. (F) Expressions of EMT-related markers of indicated SMMC7721 cells were detected by RT-PCR. “N.S.” indicated not significant; asterisk indicated P < 0.050, all in vitro experiments were performed in triplicate, and t tests were used. (TIF 4551 kb) [file 13045_2019_724_MOESM8_ESM.tif]

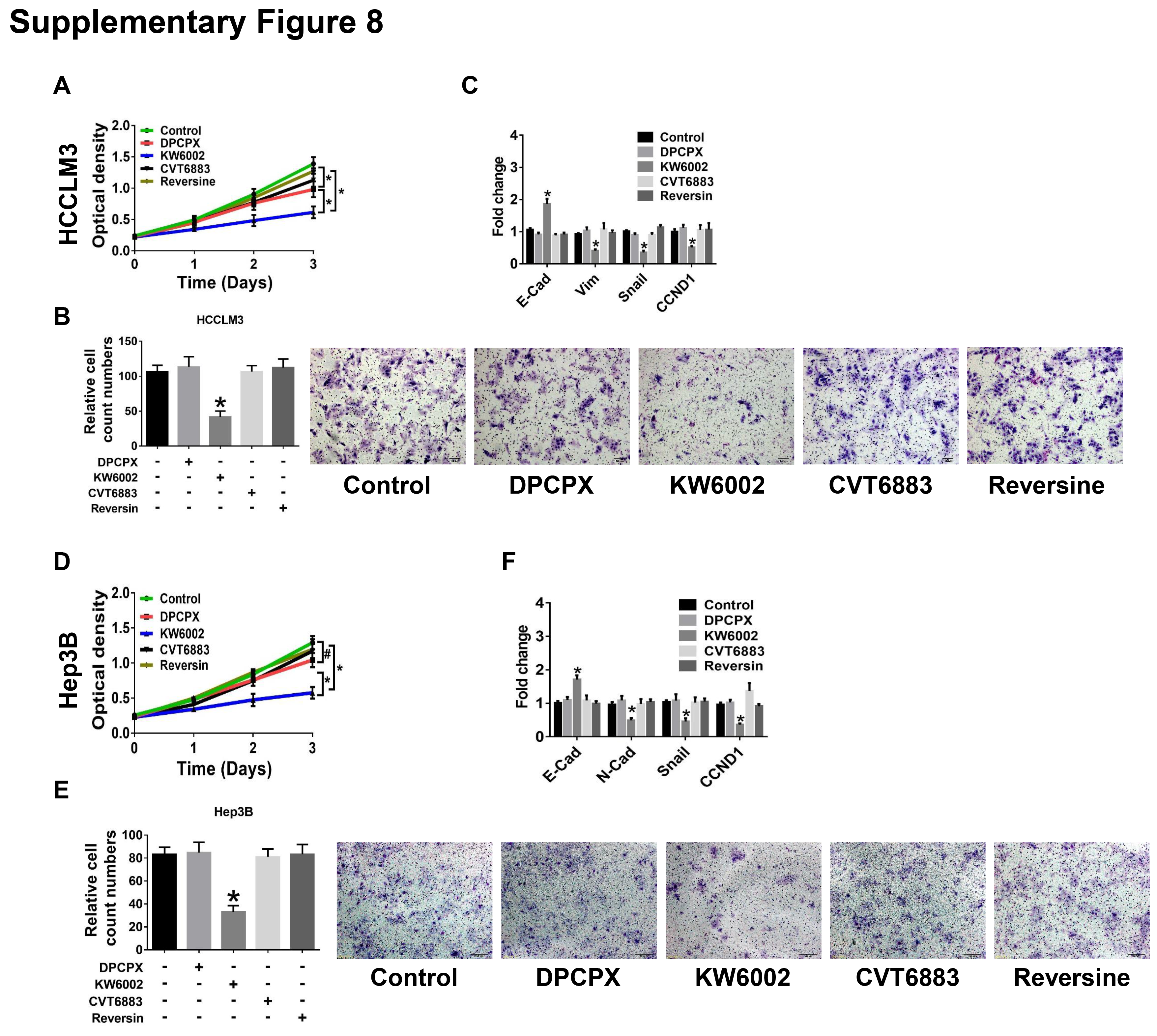

Supplement: Supplementary file 9 — Figure S8. CD73 function in HCC occurs through adenosine receptor A2A. (A) Proliferation capacity in HCCLM3 cells treated with indicated adenosine receptor antagonists detected by CCK-8 assays. (B) Invasion capacity in HCCLM3 cells treated with indicated adenosine receptor antagonists detected by Transwell assays. (C) Expressions of EMT-related markers in HCCLM3 cells treated with indicated adenosine receptor antagonists were detected by RT-PCR. (D) Proliferation capacity in Hep3B cells treated with indicated adenosine receptor antagonists detected by CCK-8 assays. (E) Invasion capacity in Hep3B cells treated with indicated adenosine receptor antagonists detected by Transwell assays. (F) Expressions of EMT-related markers of Hep3B cells treated with indicated adenosine receptor antagonists were detected by RT-PCR. Asterisk indicated P < 0.050, all in vitro experiments were performed in triplicate, and t tests were used. (TIF 5733 kb) [file 13045_2019_724_MOESM9_ESM.tif]

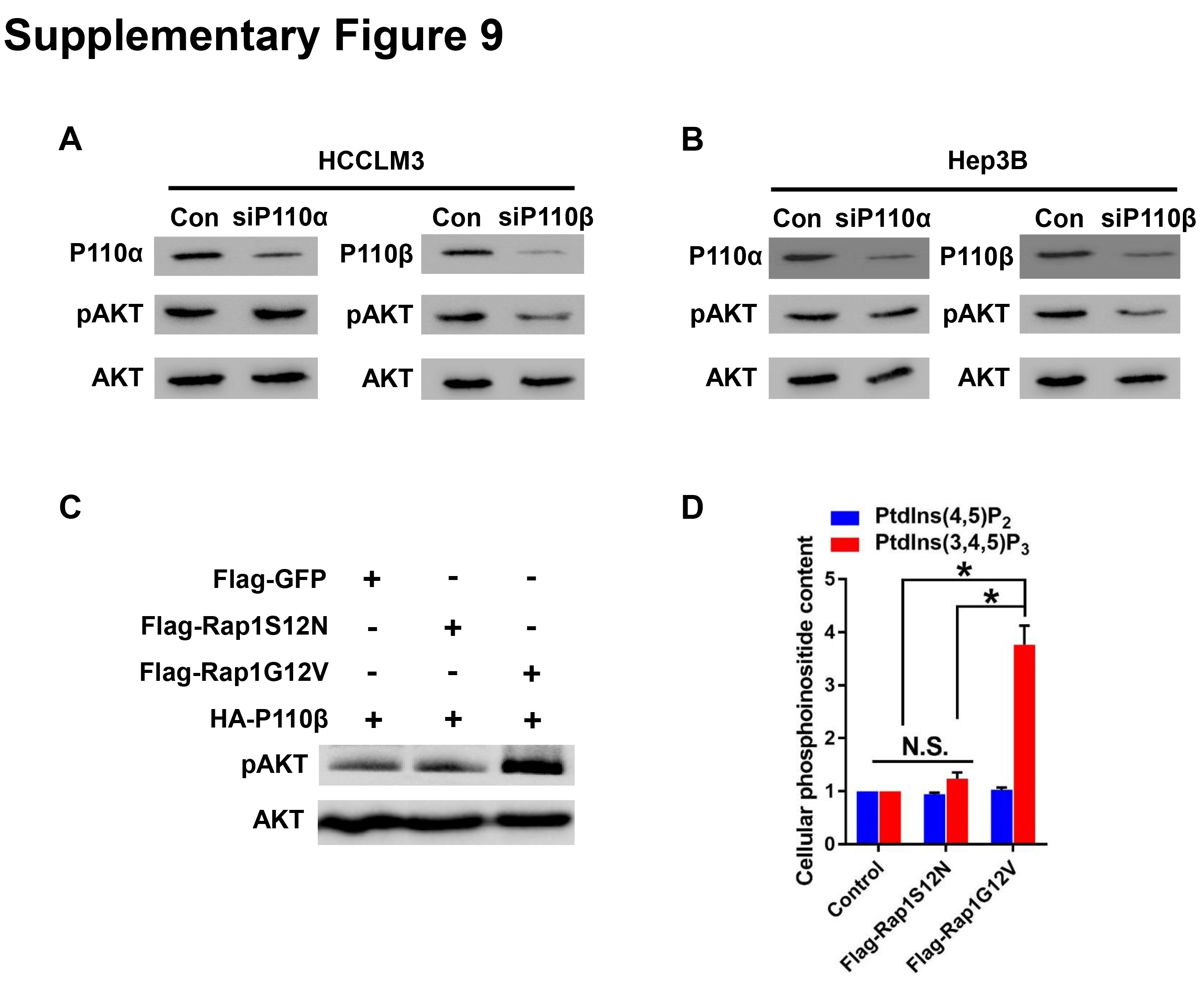

Supplement: Supplementary file 10 — Figure S9. Role of Rap1 activation in AKT phosphorylation. (A) Expressions of P110α, P110β, pAKT, and AKT in HCCLM3 cells were detected by WB assays. (B) Expressions of P110α, P110β, pAKT, and AKT in Hep3B cells were detected by WB assays. (C) Expressions of pAKT and AKT in 293T transfected with indicated plasmids were detected by WB assays. (D) Cellular PIP2 and PIP3 levels of 293T transfected with indicated plasmids were detected by ELISA assays. “N.S.” indicated not significant; asterisk indicated P < 0.050, experiments were performed in triplicate, and t tests were used. (TIF 725 kb) [file 13045_2019_724_MOESM10_ESM.tif]

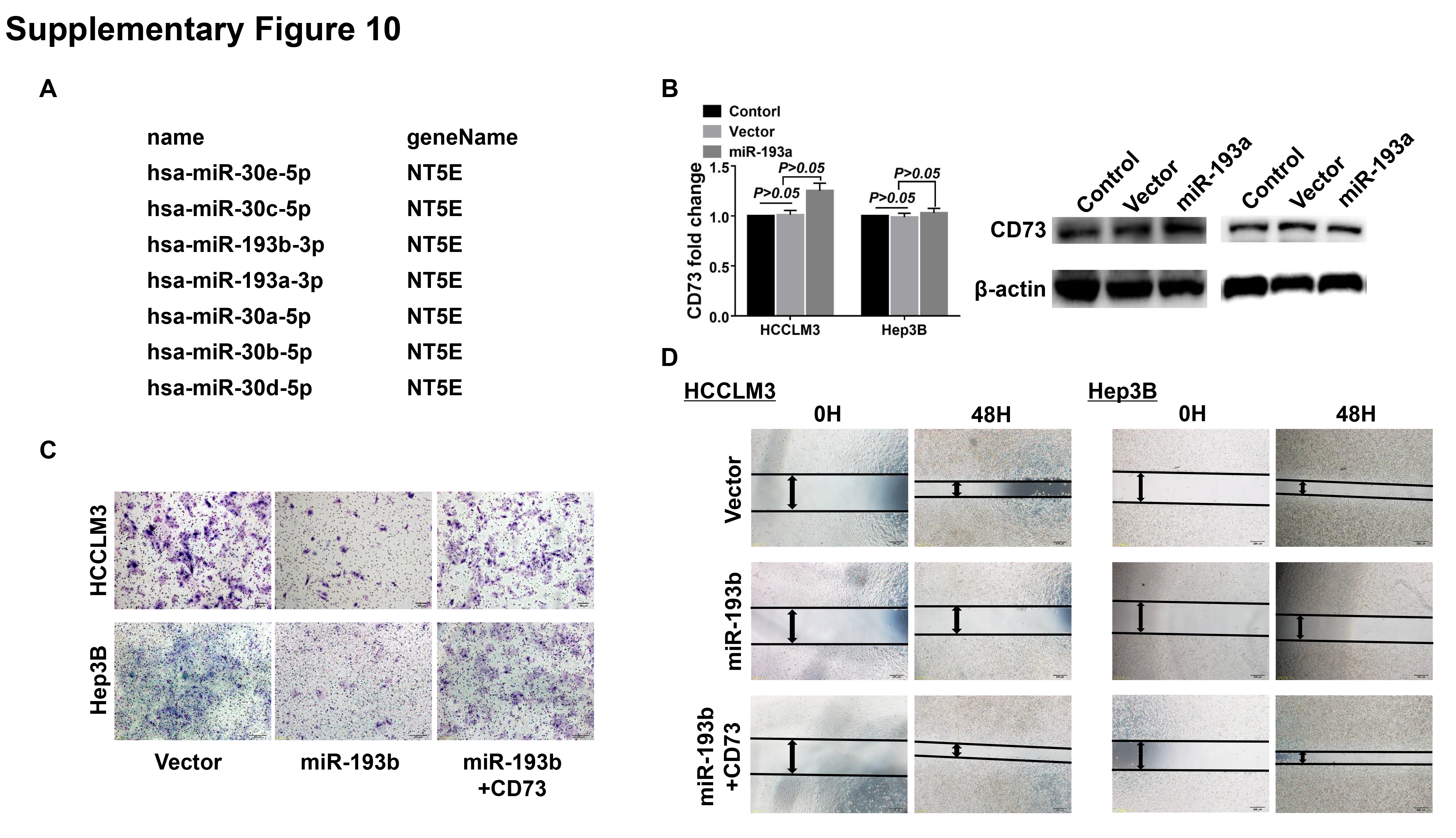

Supplement: Supplementary file 11 — Figure S10. miR-193b served as an upstream negative regulator for CD73 expression in HCC. (A) Prediction results of microRNAs that potentially regulate CD73 according to StarBase 2.0. (B) Effects of miR-193a mimics transfection on CD73 expression in HCC cells were evaluated by RT-PCR and WB assays. (C) Representative images of Transwell assays in indicated HCCLM3 (upper) and Hep3B (lower) cells. (D) Migration evaluation via wound healing assays of miR-193b mimic-transfected HCC cells with or without CD73 overexpression. All in vitro experiments were performed in triplicate, and t tests were used. (TIF 6462 kb) [file 13045_2019_724_MOESM11_ESM.tif]

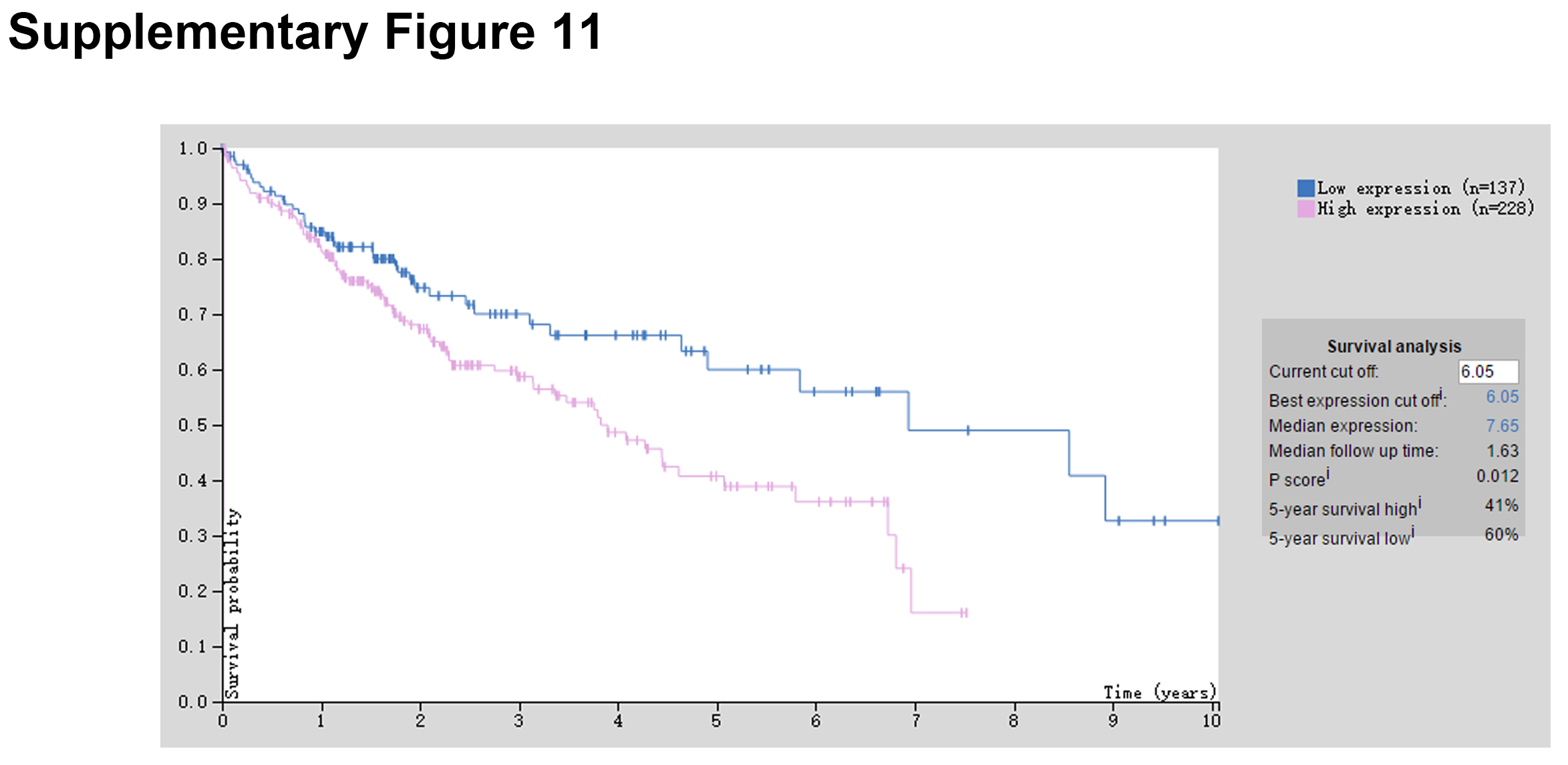

Supplement: Supplementary file 12 — Figure S11 Clinical significance of CD73 in HCC according to TCGA database. Data was collected from The Human Protein Atlas (https://www.proteinatlas.org/ENSG00000135318-NT5E/pathology/tissue/liver+cancer). (TIF 378 kb) [file 13045_2019_724_MOESM12_ESM.tif]
